# Development of an orally available inhibitor of CLK1 for skipping a mutated dystrophin exon in Duchenne muscular dystrophy

**DOI:** 10.1038/srep46126

**Published:** 2017-05-30

**Authors:** Yukiya Sako, Kensuke Ninomiya, Yukiko Okuno, Masayasu Toyomoto, Atsushi Nishida, Yuka Koike, Kenji Ohe, Isao Kii, Suguru Yoshida, Naohiro Hashimoto, Takamitsu Hosoya, Masafumi Matsuo, Masatoshi Hagiwara

**Affiliations:** 1 Department of Anatomy and Developmental Biology, Kyoto University Graduate School of Medicine, Kyoto, Japan; 2 Medical Research Support Center, Kyoto University Graduate School of Medicine, Kyoto, Japan; 3 Department of Medical Rehabilitation, Faculty of Rehabilitation, Kobegakuin University, Kobe, Japan; 4 Laboratory of Chemical Bioscience, Institute of Biomaterials and Bioengineering, Tokyo Medical and Dental University, Tokyo, Japan; 5 Department of Regenerative Medicine, National Center for Geriatrics and Gerontology, Oobu, Japan

## Abstract

Duchenne muscular dystrophy (DMD) is a fatal progressive muscle-wasting disease. Various attempts are underway to convert severe DMD to a milder phenotype by modulating the splicing of the *dystrophin* gene and restoring its expression. In our previous study, we reported TG003, an inhibitor of CDC2-like kinase 1 (CLK1), as a splice-modifying compound for exon-skipping therapy; however, its metabolically unstable feature hinders clinical application. Here, we show an orally available inhibitor of CLK1, named TG693, which promoted the skipping of the endogenous mutated exon 31 in DMD patient-derived cells and increased the production of the functional exon 31-skipped dystrophin protein. Oral administration of TG693 to mice inhibited the phosphorylation of serine/arginine-rich proteins, which are the substrates of CLK1, and modulated pre-mRNA splicing in the skeletal muscle. Thus, TG693 is a splicing modulator for the mutated exon 31 of the dystrophin gene *in vivo*, possibly possessing therapeutic potential for DMD patients.

Duchenne muscular dystrophy (DMD) is an X-linked inherited neuromuscular disorder that affects approximately 1 out of 3,500 newborn boys, and the most prevalent fatal muscle-wasting disease^1^,^2^,^3^. It is characterized by progressive muscle deterioration and wasting due to the absence of dystrophin^3^. Unfortunately, DMD patients usually succumb to cardiac or respiratory failure attributed to muscle dysfunction in their 20s, and no effective cure is currently available. Nonsense mutations or deletions of exons, which cause frame shifts in the dystrophin mRNA and generate premature termination codons (PTCs), usually result in the lack of dystrophin protein and development of the disease.

Current therapeutic attempts to restore dystrophin protein expression include the induction of exon skipping with antisense oligonucleotides (AONs)^4^,^5^,^6^,^7^ or small molecules (TG003)^8^ and the suppression of nonsense-mediated mRNA decay, which includes the read-through of PTC (e.g. Ataluren)^9^,^10^, by chemical compounds. Several AONs have been designed as exon-skipping therapies, in which ribosomes “skip” the mutated exons in pre-mRNA transcripts to produce an in-frame, but truncated, transcript that is translated into a functional dystrophin protein^11^,^12^,^13^. However, considering their therapeutic cost and delivery efficiency to muscles, small chemical compounds with exon-skipping function are highly anticipated for DMD therapy.

We previously reported on the small molecule TG003 that promotes skipping of dystrophin exon 31 in cells harboring the c.4303G > T mutation^8^. In the patient’s muscle cells, two dystrophin mRNA transcripts of different lengths are detected in nearly equal amounts—one with the premature TAG termination codon in exon 31, and the other lacking only exon 31. The dystrophin mRNA lacking the 111-bp exon 31 is in-frame to produce an internally deleted, but functional dystrophin protein. The expression of the functional truncated protein is enhanced by TG003 treatment.

Our initial findings showed that TG003 is an inhibitor of CDC-like-kinases (CLKs; CLK1, 2, 4)^14^, which phosphorylate stretches of alternating serine and arginine residues in serine/arginine-rich (SR) protein family members^15^,^16^. CLK-dependent phosphorylation of SR proteins induces their association with transcribed RNAs^17^,^18^,^19^ to promote or, in some cases, repress splice-site recognition, resulting in alternative splicing^20^,^21^,^22^. Thus, TG003 modifies mRNA splicing by inhibiting the phosphorylation of SR proteins^14^,^23^,^24^.

Pharmacological efficacy in target tissues is an unavoidable issue for developing therapeutic modalities. While TG003 was effective in modulating CLK activity and mRNA splicing in cellular models of DMD^8^, its metabolic instability hinders its clinical application. In this study, we identified an orally available selective CLK1 inhibitor, TG693, which promoted exon skipping and production of a truncated functional dystrophin protein in immortalized cells derived from a patient with DMD (c.4303G > T) to the same extent as TG003. Additionally, TG693 was metabolically stable and transiently detected in the skeletal muscle at a bioactive level, supporting its therapeutic potential as an orally available drug candidate.

## Results

### Development of the metabolically stable CLK1 inhibitor TG693

**Figure 1:**
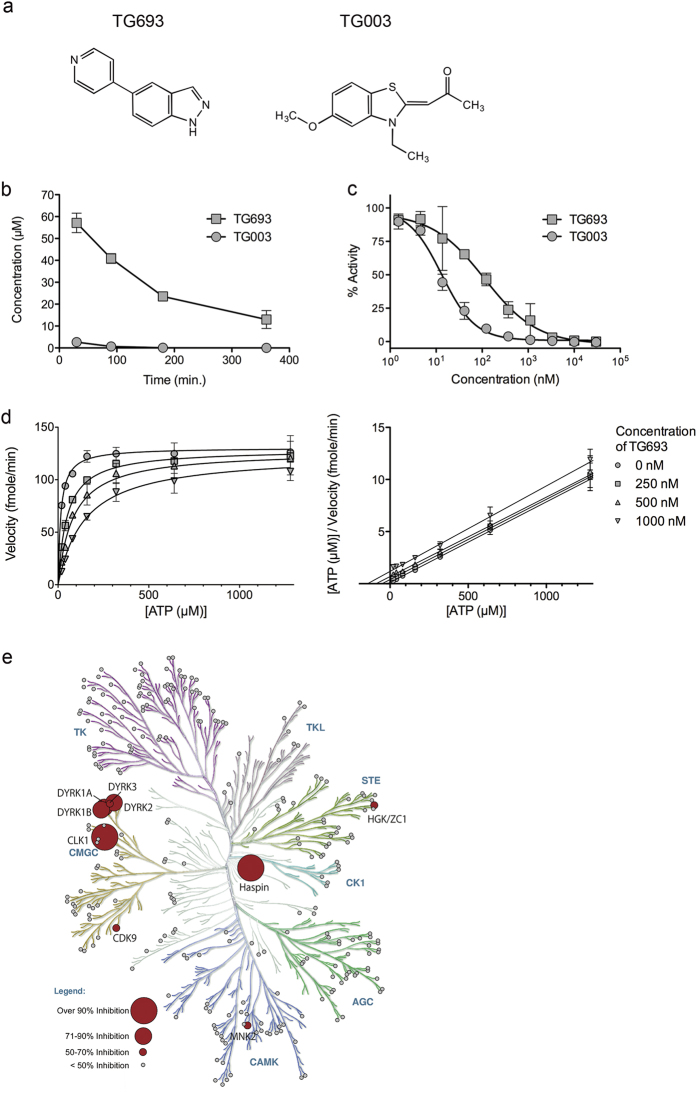
TG693 is a metabolically stable CDC2-like kinase 1 (CLK1) inhibitor. (**a**) TG693 and TG003 chemical structures. (**b**) Pharmacokinetic profile of TG693 after a single 30 mg kg^−1^ dose administered by subcutaneous injection in imprinting control region (ICR) mice. Data indicate the mean ± SEM (n = 3). (**c**) Recombinant CLK1 was incubated with the substrate peptide in the presence of the indicated concentrations of small molecules. Data represent the means ± SD (n = 3). Representative dose-response curves with Hill slopes are shown. (**d**) TG693 competitive ATP inhibition is shown in Michaelis-Menten (left) and Hanes-Woolf (right) plots. CLK1 kinase activity was measured at the indicated concentrations of TG693 and ATP. Velocity was plotted versus [ATP] and [ATP]/velocity was plotted versus [ATP]. (**e**) Map of the inhibitory activities of TG693 on a kinase dendrogram. Percent inhibition by 1 μM TG693 was measured for a panel of 313 kinases. Red circles indicate the inhibited kinases and are sized according to percent inhibition. The illustration was reproduced courtesy of Cell Signaling Technology, Inc. (www.cellsignal.com).

We screened for stable exon-skipping inducers of the mutant DMD exon 31 (c.4303G > T) in our original chemical library and identified the small molecule 5-(4-pyridinyl)-1*H*-indazole (henceforth referred to as TG693) as an exon-skipping inducer (Fig. 1a). To test the *in vivo* stability of TG003 and TG693, mice were administered a single subcutaneous injection of either compound and then serum concentrations were monitored by liquid chromatography-mass spectrometry (LC/MS) at serial time points. While only trace amounts of TG003 were detected, the average TG693 serum concentration was 13 μM at 6 h post-injection, indicating that TG693 was more stable than TG003 in the blood plasma (Fig. 1b). Subsequent analyses on CLK1 inhibition with *in vitro* kinase assays revealed that TG693 inhibited the phosphorylation of a synthetic SRSF1 RS domain peptide with a half-maximal inhibitory concentration (IC_50_) of 112.6 nM, approximately 10-fold weaker than that of TG003 (IC_50_ = 13.1 nM) (Fig. 1c). We also examined whether TG693 served as an ATP-competitive inhibitor. Hanes-Woolf plots showed parallel lines with y-intercepts that correspond to TG693 concentration, demonstrating that TG693 competes with ATP for a single site in CLK1 (Fig. 1d; *K*_*m*_ = 14.88 μM, *K*_*I*_ = 105.3 nM). Moreover, kinetic parameters (*K*_*m*_^apparent^, *V*_*max*_^apparent^, α, α’) were determined by Michaelis-Menten model fitting (Supplementary Table S1), which indicated that TG693 was an ATP-competitive type I inhibitor that antagonized CLK1. Further, in order to evaluate the specificity of TG693-mediated kinase, we investigated the *in vitro* inhibitory activity of 1 μM TG693 against a panel of 313 recombinant kinases. TG693 inhibited CLK1 and Haspin activity by over 90% and kinases over 50% inhibition were shown in a kinase dendrogram (Fig. 1e). These suggested that TG693 was a potent and selective inhibitor of CLK1.

### TG693 inhibits CLK1 kinase activity in cells

**Figure 2:**
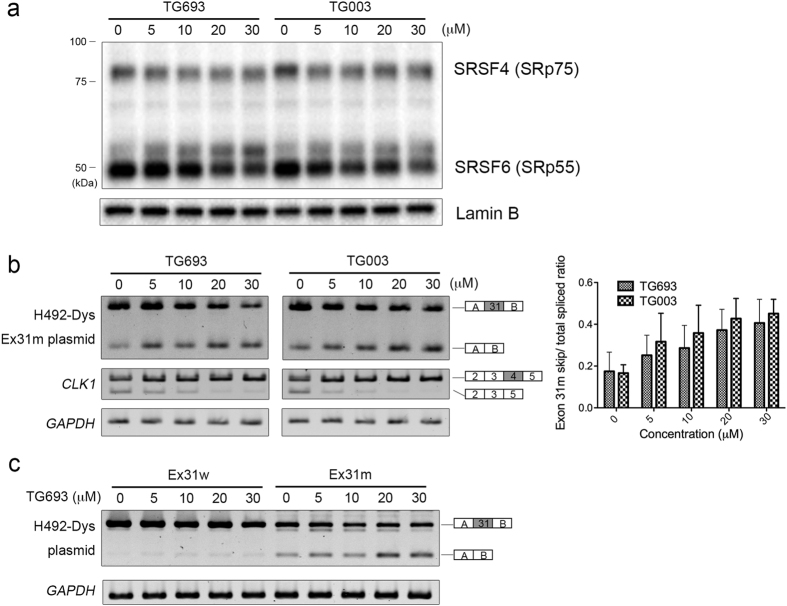
TG693 promotes skipping of mutated exon 31 in HeLa cells in a dose-dependent manner. (**a**) SR protein phosphorylation was assessed in HeLa cells treated with TG693 and TG003 for 1 h. Lamin B served as a loading control. Uncropped images have been provided in Supplementary Fig. S4. (**b**,**c**) Effect of TG693 on exon 31 skipping with the reporter plasmid. Transfected HeLa cells were incubated in the presence of TG693, TG003, or DMSO vehicle for 24 h. Reporter and endogenous *CLK1* splicing was then analyzed by RT-PCR. *GAPDH* served as a control. The Splicing ratios were quantified by intensity analysis and normalized to *GAPDH* expression. Uncropped images have been provided in Supplementary Figs S5 and S6, respectively. Data represent the means ± SD (n = 3).

TG003 administration to cultured cells inhibits the CLK1-mediated phosphorylation of SR proteins, particularly that of SRSF4^17^. To examine the cellular activity of TG693, HeLa cells were treated with increasing concentrations of compounds, and then SR protein phosphorylation was assessed by western blotting with an anti-pan-phospho-SR antibody that recognizes canonical SR protein family members^25^. Notably, TG693 and TG003 inhibited the phosphorylation of SRSF4 at 5 μM and SRSF6 at 20 μM and 10 μM, respectively (Fig. 2a), confirming that these compounds act via the same biochemical mechanism.

### TG693 induces exon-skipping of a DMD splicing reporter

Since TG693 was shown to be a metabolically stable CLK1 inhibitor, we evaluated the dose-dependent effects of TG693 on the skipping of mutant DMD exon 31 (c.4303G > T) with HeLa cells expressing a H492-Dys Ex31m splicing reporter^8^. Interestingly, the effects of TG693 and TG003 on the exon-skipping were comparable, despite their differences in suppressing CLK1 activity in *in vitro* kinase assays (Fig. 2b, upper panel); and TG693 had no effect on wild type transcript splicing even at 30 μM (Fig. 2c). To determine if this activity was specific to exon 31 mutation, we then evaluated the function of TG693 on exon-skipping with the H492-dysEx27m splicing reporter containing a point deletion in exon 27 (c.3613delG (p.Glu1205LysfsX9)) that causes a frameshift-induced premature termination codon. As shown in Supplementary Fig. S1, TG693 induced exon 27-skipping from H492-dysEx27m reporter, similar to that previously observed with TG003^8^.

*CLK1* pre-mRNA splicing is regulated by CLK1 kinase activity^14^. In this mechanism, abundant CLK1 levels induce SR phosphorylation, resulting in the removal of exon 4 from *CLK1* pre-mRNA and nonsense-mediated mRNA decay^26^,^27^. In previous reports, administration of TG003 to cultured cells promoted full-length *CLK1* expression^14^,^17^. Consistently, *CLK1* exon 4-skipped transcript expression was attenuated in the presence of 5 μM TG693 (Fig. 2b, middle panel), suggesting that TG693 exhibited a considerable inhibitory effect on CLK1 and concomitant exon-skipping, similar to that of TG003^8^.

### TG693 restored dystrophin expression in patient-derived cells

**Figure 3:**
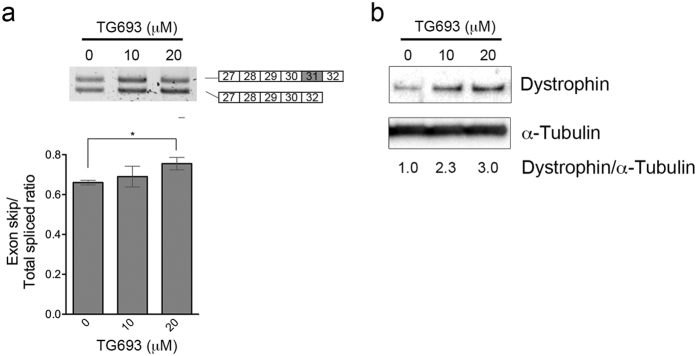
TG693 induces truncated dystrophin protein expression in patient-derived cells. Immortalized DMD patient-derived cells were treated with increasing concentrations of TG693 for 2 d. (**a**) Semi-quantitative RT-PCR for dystrophin exon skipping. The uncropped image is provided in Supplementary Fig. S7. Data represent the means ± SD (n = 3). *p < 0.05. (**b**) Western blotting of dystrophin protein expression in TG693-treated cells using C-terminal-directed antibody. α-Tubulin was used as a loading control. Uncropped images have been provided in Supplementary Fig. S8. Data are representative of three independent experiments.

We previously reported that TG003 restored dystrophin production in patient-derived myotubes^8^. Therefore, we investigated the effect of TG693 on dystrophin expression in an immortalized cell line derived from a DMD patient with the c.4303G > T mutation, which was by established by the forced expression of constitutively active cyclin-dependent kinase 4, cyclin D1, and telomerase^28^,^29^. Notably, TG693 promoted the skipping of mutant exon 31 (Fig. 3a), but did not affect any other exons (Supplementary Fig. S2 and Table S4). Moreover, TG693 was sufficient to increase dystrophin protein expression in a dose-dependent manner with a 130% and 200% increase at 10 and 20 μM, respectively (Fig. 3b). Thus, these results supported the therapeutic potential for TG693 in DMD patient cells harboring the c.4303G > T mutation in dystrophin exon 31.

### Oral administration of TG693 modulated splicing in mouse skeletal muscle

**Figure 4:**
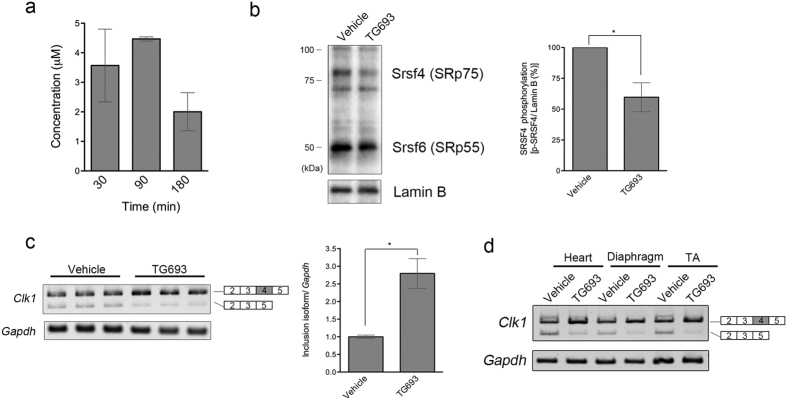
TG693 effect as a CLK1 inhibitor in the mouse tibialis anterior muscle. (**a**) TG693 bioavailability in the tibialis anterior (TA) muscle of ICR mice after oral administration of a single 30 mg kg^−1^ dose. Data represent the mean ± SEM (n = 3). (**b**) SR protein phosphorylation status in the TA muscle of ICR mice after oral administration. Lamin B served as a loading control. SRSF4 phosphorylation was quantified by densitometry. Uncropped images are provided in Supplementary Fig. S9. Data represent means ± SD (n = 5). *p < 0.05. (**c,d**) *Clk1* expression in the TA muscle, heart and diaphragm were analyzed by RT-PCR with a *GAPDH* internal control. Uncropped images are provided in Supplementary Fig. S10 and in Supplementary Fig. S11, respectively. Data represent the means ± SD (n = 3). *p < 0.05.

To analyze the *in vivo* effects of TG693, mice were orally administered TG693 (30 mg kg^−1^). Notably, a significant amount of TG693 (>4 μM) was detected in the tibialis anterior (TA) muscle of mice (Fig. 4a), as well as a marked concomitant reduction of SR protein phosphorylation, particularly that of SRSF4 (Fig. 4b). Further analysis of splicing modulation showed that TG693 promoted full-length *Clk1* transcript expression in TA, suggesting that treatment suppressed the excision of exon 4 induced by CLK1 activity (Fig. 4c). TG693 was active in other muscle tissues, such as the heart and diaphragm, as well as skeletal muscle (Fig. 4d). We also confirmed that TG693 had no apparent toxicity in rats at up to 100 mg kg^−1^ *per os* (Supplementary Fig. S3). No mortalities or gross abnormalities were observed in TG693-treated animals during the experimental period, nor did it affect body weight in mice administrated an overdose of the compound. Thus, these results suggest that TG693 is a useful tool for inhibition of CLK1 activity *in vivo per os*.

## Discussion

We succeeded in identifying a selective ATP-competitive inhibitor of CLK1, named TG693. TG693 is structurally distinct from TG003 (Fig. 1a), but it similarly shows potent CLK1 inhibition as well as dystrophin exon 31 skipping enhancement in cells (Fig. 2a and b), strengthening our original hypothesis that CLK1 can be the molecular target for splicing manipulation^8^,^14^. TG693 restored the dystrophin protein expression in cells harboring a point mutation (c.4303G > T) in exon 31 of the dystrophin gene by inducing exon-skipping (Fig. 3). The c.4303G > T point mutation in exon 31 attenuates exon recognition by disruption and creation of splicing elements such as exonic splicing enhancer and silencer^8^,^30^,^31^. Thus, mutated exon 31 is targeted by TG693 to suppress exon recognition without altering wild type transcript splicing (Fig. 2c). We also showed TG693 promotes skipping of a mutated exon 27 in another patient (Supplementary Fig. S1). *In vivo* mouse experiments revealed that oral TG693 administration decreased SR protein phosphorylation in muscle tissue and modulated the splicing pattern of endogenous *Clk1* (Fig. 4). Thus, these results indicated that TG693 could modulate the splicing pattern of the mutated dystrophin gene in the patient by oral administration. This represents a significant advance in drug-induced exon skipping therapy for DMD.

Splicing modulation has drawn increasing attention as a therapeutic strategy for DMD. In 2016, two antisense drugs that modulate splicing were approved by the U.S. Food and Drug Administration (FDA) (e.g. Eteplirsen and Nusinersen), and exon-skipping therapy has become a reality. While AONs offer a specific way to modulate splicing in a therapeutic manner, some hurdles must be overcome for the orally available antisense drugs^32^,^33^. Compared to AONs, small chemical compounds may not be as specific, but more advantageous for the synthesis cost and the oral availability. Thus, splicing modulation with small molecules can become an alternative therapeutic approach in addition to AONs, and can expand applications of splicing modulation therapy.

CLK1 inhibitors have attracted extensive attention in recent years. Notably, TG693 is the first and only CLK1 inhibitor which is orally available and metabolically stable in blood^34^,^35^. As such, we believe that the present study will provide a foundation for the continued development of therapies other diseases associated with aberrant CLK1 activity—such as Alzheimer’s and Influenza^36^,^37^.

Our results show that TG693 mediated CLK1 inhibition elicits changes in SR protein phosphorylation, particularly that of SRSF4 and SRSF6 (Figs 2a and 4b). SR proteins regulate a multitude of splicing events in the phosphorylation dependent manner^21^, raising the possibility that reduction of SR proteins phosphorylation may cause off-target splicing alteration. Therefore, we previously checked transcriptional wide effect of TG003, which similarly inhibits phosphorylation of SR proteins as TG693 *in vitro* (Fig. 2a), and we found that only 0.3% (110/37487)^24^ of registered alternative splicing events were skipped enhanced by TG003. TG693 is fairly specific in its inhibition profile, showing significant off target effects (>70%) to only 4 of 313 kinases (Fig. 1e). This solidifies the mode-of-action evidence for TG693. Although we confirmed TG693 had no apparent acute toxicity in rats at up to 100 mg kg^−1^ *per os* (Supplementary Fig. S3), more preclinical studies with other animal models are necessary to consider the clinical application of TG693. We have also started a project to seek next generation CLK1 inhibitors with a pharmaceutical company to improve the efficacy and selectivity of TG693 based on the information written here.

TG693 is a simple compound with indazole and pyridine groups (Fig. 1a) far more metabolically stable than TG003 which is a benzothiazole compound. We believe that TG003 is easily metabolized via *O*-demethylation and subsequent sulfate conjugation of the hydroxyl group^38^ and likely interacts with CLK1 via hydrogen bonds in the ATP-binding pocket^39^. In the case of TG693, metabolically stable indazole moiety is suspected to form a hydrogen bond with an amino acid residues of CLK1^40^, but structural studies will be necessary to confirm this hypothesis.

Our results suggest that exon-skipping therapy with orally available small molecule has therapeutic potential to genetic diseases caused by disruption/creation of splicing elements, including Duchenne muscular dystrophy. We believe that our approach is potentially applicable to other genetic diseases caused by nonsense or frameshift mutations in the internal coding exons whose length is a multiple of three. On this criteria, we found 6,244 pathogenic mutations in the NIH ClinVar database that may be target candidates for exon-skipping therapy with TG693. Our efforts have focused on expanding application of TG693 to other diseases, addition to the Duchenne muscular dystrophy.

## Methods

### Materials

TG693 was synthesized as detailed in the Supporting Information. TG003 was prepared as described previously^14^. Both compounds were dissolved in dimethylsulfoxide (DMSO) (Hybri-MAX^TM^, Sigma-Aldrich, St. Louis, MO, USA). Carboxymethyl cellulose sodium salt was obtained from Nacalai Tesque (Kyoto, Japan).

### Antibodies

The source and catalog numbers of commercially available primary antibodies have been provided in Supplemental Table 2. Mouse monoclonal antibodies against phospho-SR proteins (1H4) and α-tubulin (DM1A) were purchased from Invitrogen (Carlsbad, CA, USA) and Thermo Fisher Scientific (Yokohama, Japan), respectively. Rabbit polyclonal antibody against dystrophin was obtained from Abcam (Cambridge, UK). Goat polyclonal antibody against Lamin B (M-20) was purchased from Santa Cruz Biotechnology (Santa Cruz, Dallas, TX, USA). HRP-conjugated anti-rabbit, anti-goat IgG, and anti-mouse IgG secondary antibodies were purchased from GE Healthcare Life Sciences (Pittsburgh, PA, USA), Jackson ImmunoResearch (West Grove, PA, USA), and Abcam, respectively.

### *In vitro* kinase assay

CLK1 kinase activity was assessed as previously reported^14^ with minor modifications. The reaction mixture containing serially diluted inhibitors, 10 mM MOPS-KOH (pH 6.5), 10 mM magnesium chloride, 200 μM EDTA, 1 μM ATP, 0.167 μCi of [γ-^32^P] ATP, 0.417 μg of synthetic RS peptide, and recombinant GST-tagged human CLK1 (Cat #04–126, Carna Biosciences, Kobe, Japan) was prepared in a final volume of 25 μL. The reaction mixture was incubated at 30 °C for 30 min, and phosphoric acid was added at a final concentration of 5% to stop the reaction. Then, 25 μL of the reaction mixture was dispensed on a P81 phosphocellulose membrane (Cat #20-134, Merck Millipore, Darmstadt, Germany) and washed four times in 5% phosphoric acid. Cherenkov light from the incorporated ^32^P was measured using a liquid scintillation counter. The kinase assay conditions, including the incubation period and concentrations of kinases and substrates, were optimized to maintain linearity during incubation. The net radioactivity was determined by subtracting the background count from the reaction mixture without kinases. The IC_50_ of each compound was calculated by interpolation on a log-concentration-response curve fitted with a four-parameter logistic equation.

### Kinase profiling

The effect of 1 μM TG693 on 313 kinases (listed in Supplementary Table S2) was examined with immobilized metal ion affinity-based fluorescence polarization (IMAP) assay or off-chip mobility shift assay (MSA) depending on the kinase being examined with an ATP concentration at the *K*_*m*_ or 1 mM. These assays rely on the phosphorylation of small synthetic peptides developed for each kinase, and detect activity by changes in electrophoretic mobility (MSA assay) or by binding of a fluorescently labelled peptide to a large micro-particle and concomitant polarization increases (IMAP assay). Reaction conditions for each kinase are described in Supplementary Table S5. All kinase assays were carried out at Carna Biosciences (Kobe, Japan). The inhibitory map was constructed using Kinome Render^41^.

### Immobilized metal ion affinity-based fluorescence polarization (IMAP) Assay

Substrate/ATP/metal (4×) and kinase (2×) solutions were prepared using with assay buffer (20 mM HEPES, 0.01% Tween-20, 2 mM DTT, pH 7.4), and then mixed in 384-well black plates for 1 hour at room temperature. IMAP binding solution (IMAP Screening Express kit; Molecular Devices, Sunnyvale, CA, USA) was added to each well and incubated for 30 min. The level of kinase activity was then evaluated by fluorescence polarization at 485/530 nm (Ex/Em).

### Off-Chip Mobility Shift Assay (MSA)

Inhibitor (4×), substrate/ATP-Metal (4×), and kinase (2×) solutions were prepared with assay buffer (20 mM HEPES, 0.01% Triton X-100, 2 mM DTT, pH 7.5) and mixed in 384-well plates at room temperature for 1 or 5 h depending on the kinase. Reactions were stopped by the addition of termination buffer (QuickScout Screening Assist MSA; Carna Biosciences). The entire reaction mixture was then applied to a LabChip system (Perkin-Elmer, Waltham, MA, USA) and the product and substrate peptide peaks were separated and quantified, and then heights of product (P) and substrate (S) peptide peaks were calculated. The level of kinase activity was evaluated with the following equation: activity = P/(P + S).

### ATP kinetics assay

Kinetic reactions were performed with the same method used for *in vitro* kinase assays, except that 0.05–0.4 μCi of [γ-^32^P] ATP was used. *K*_m_ and *K*_i_ values were calculated with a competitive inhibition model in Prism 6 software (GraphPad Software, San Diego, CA, USA).

### Plasmid construction, cell culture, and transfection

The H492-dys Ex31m, H492-dys Ex31w, and H492-dys Ex27m plasmids were prepared as described previously^8^. HeLa cells were maintained in low-glucose Dulbecco’s modified Eagle’s medium (Nacalai Tesque) supplemented with 10% fetal bovine serum, 100 U/mL penicillin, and 100 μg/mL streptomycin. Immortalized muscle cells (D4P4) from a DMD patient with the c.4303G > T mutation were established as described previously^28^. Cells were maintained in Dulbecco’s modified Eagle’s medium (Wako Pure Chemical Industries, Osaka, Japan) supplemented with 20% fetal bovine serum (Gibco, Grand Island, NY, USA), 2% Ultroser™ G serum substitute (Pall Corp., NY, USA), and 1% antibiotic-antimycotic solution (Gibco). Transfections were carried out using Lipofectamine 2000 (Thermo Fisher Scientific) following the manufacturer’s instructions. For reporter assays, TG693 and TG003 were added to the culture medium for 24 h before analysis.

### RNA Isolation and Semiquantitative RT-PCR

Total RNA was isolated from HeLa cells in 800 μL Sepasol-RNA I Super G (Nacalai Tesque) and then treated with RQ1 RNase-free DNase (Promega, Madison, Wisconsin, USA) according to the manufacturer’s protocol. DMD patient-derived cells were lysed in 800 μL Trizol reagent (Thermo Fisher Scientific) and then RNA was isolated with a Direct-zol RNA MiniPrep kit (Zymo Research, Irvine, CA, USA). Tibialis anterior (TA) muscle homogenates were prepared using a mortar and then RNA was isolated with an RNeasy MiniPrep kit (Qiagen, Hilgen, Germany). First-strand cDNA was synthesized using Superscript II reverse transcriptase (Invitrogen) and random hexamers (Takara Bio, Inc., Shiga, Japan). Semi-quantitative RT-PCR was performed with Ex Taq polymerase (Takara Bio, Inc.) with the following cycle conditions: 95 °C for 2 min, followed by 32 (reporter and *Clk1*) or 25 cycles (*GAPDH*) of denaturation at 95 °C for 20 s, annealing at 58 °C for 20 s, and elongation at 72 °C for 1 min, with a final 5 min incubation at 72 °C in a PCR thermal cycler (Bio-Rad Laboratories, Hercules, CA, USA). PCR products were separated by electrophoresis and stained with ethidium bromide. Images were obtained with a ChemiDoc^TM^ MP Imaging System (Bio-Rad) and analyzed with Image Lab software (Bio-Rad). Skipping efficiencies were determined by quantifying the skipped products with a DNA 1000 LabChip Kit on an Agilent 2100 Bioanalyzer (Agilent Technologies, Santa Clara, CA, USA). Exon skip/inclusion ratios were calculated as the amount of skipped transcript relative to the sum the full-length and skipped transcript. The primers for semi-quantitative RT-PCR were as follows: human *CLK1* forward, 5′-ATG AGA CAC TCA AAG AGA ACT TAC TG-3′; human *CLK1* reverse, 5′-CTT TAT GAT CGA TGC ACT CCA C-3′; mouse *Clk1* forward, 5′-ATG AGA CAT TCA AAG AGA ACT TAC TG-3′; mouse *Clk1* reverse, 5′-CAC TTT ATG ATC GAT GCA TTC C-3′; *GAPDH* forward, 5′-CCA TCA CCA TCT TCC AGG AGC GAG-3′; *GAPDH* reverse, 5′-GTG ATG GCA TGG ACT GTG GTC ATG-3′; *dystrophin* forward, 5′-CCT GTA GCA CAA GAG GCC TTA-3′; *dystrophin* reverse, 5′-TCC ACA CTC TTT GTT TCC AAT G-3′; DMD splicing reporter forward, 5′-ATT ACT CGC TCA GAA GCT GTG TTG C-3′; and DMD splicing reporter reverse, 5′-AAG TCT CTC ACT TAG CAA CTG GCA G-3′.

### Western blot analysis

Proteins were extracted from HeLa cells, immortalized DMD cells, and mouse muscle tissue using CelLytic M (Sigma-Aldrich, St. Louis, MO, USA), 1× Cell Lysis Buffer (Cell Signaling Technology, Danvers, MA, USA), and CelLytic MT (Sigma-Aldrich), respectively. All buffers were supplemented with protease and phosphatase inhibitor cocktail (#25955 and #0757461, Nacalai). Protein concentrations were quantified with Protein Assay Reagent (Pierce by Thermo Fisher Scientific). The HeLa cell and mouse tissue lysates were mixed with Sample buffer (#0949914, Nacalai), denatured at 95 °C for 5 min, and then electrophoresed in a 10% SuperSep^TM^ Ace gel (Wako). The lysates of immortalized cells were mixed with NuPAGE LDS Sample buffer (Thermo Scientific Fisher), denatured at 70 °C for 10 min, and electrophoresed in a 3–8% NuPAGE Novex Tris-Acetate gels (Thermo Scientific Fisher). The samples were then transferred to PVDF membranes (Pall Corporation, Port Washington, NY, USA). Antibody reactions were performed with Can Get Signal^®^ Immunoreaction Enhancer Solution (Toyobo, Osaka, Japan). Peroxidase activities were visualized with ImmunoStar^®^ LD (Wako) and a ChemiDoc^TM^ MP Imaging System (Bio-Rad).

### Mice and TG693/TG003 administration

Seven-week-old, male Jcl:TCR mice were obtained from Charles River Laboratories (Shiga, Japan). All procedures were performed in accordance with the US National Institutes of Health Guide for the Care and Use of Laboratory Animals and approved by the Institutional Animal Care and Use Committee of Kyoto University Graduate School of Medicine. Mice were subcutaneously injected with 30 mg kg^−1^ TG693 or TG003 suspended in 5% DMSO, 5% Solutol, 9% Tween-80, and 81% saline. TG693 was also administered orally at a dose of 30 mg kg^−1^ suspended in 0.5% carboxymethylcellulose.

### *In vivo* pharmacokinetic analysis

TG693 metabolism and tissue bioavailability were assessed in mouse plasma and muscle tissue. To collect plasma, mice were anesthetized with isoflurane (Mylan, Osaka, Japan) at the indicated time point. Whole blood samples were collected, allowed to clot, and then centrifuged to isolate serum. TG693 or TG003 serum levels were analyzed by LC/MS with an Agilent 6420 Q-TOF mass spectrometer (Agilent Technologies) and ZORBAX HILIC Plus (TG693) or ZORBAX Eclipse Plus C18 (TG003) columns (Agilent Technologies). For muscle tissue bioavailability, mice were sacrificed by cervical dislocation and the tibialis anterior (TA) muscles, heart, and diaphragm dissected. TA homogenates were prepared using a Bead Crusher μT-12 shaking machine (Taitec, Kyoto, Japan) in saline and used to assess TG693 levels by LC/MS with a Poroshell 120 PFP (Agilent Technologies).

### Statistical analysis

Statistical analysis was performed using Student’s t-tests, except for the comparison of exon-skipping ratio in patient-derived cells analyzed by one-way ANOVA with independent *post hoc* Tukey’s multiple comparison testing. Results are reported as the means ± SD. Statistical significance was defined as *p* < 0.05. IC_50_ values were calculated from data fitted to a four-parameter logistic curve (variable slope) in Prism 6.0 software.

## Additional Information

**How to cite this article**: Sako, Y. *et al*. Development of an orally available inhibitor of CLK1 for skipping a mutated dystrophin exon in Duchenne muscular dystrophy. *Sci. Rep.* **7**, 46126; doi: 10.1038/srep46126 (2017).

**Publisher's note:** Springer Nature remains neutral with regard to jurisdictional claims in published maps and institutional affiliations.

## Supplementary Material

Supplementary Information
