# Supplementary material for: Development of an orally available inhibitor of CLK1 for skipping a mutated dystrophin exon in Duchenne muscular dystrophy
Source: Sci Rep. 2017 May 30;7:46126. doi: 10.1038/srep46126 (PMC5448077; doi:10.1038/srep46126)
Supplement: Supplementary Information [file srep46126-s1.doc]

**Supporting Information**

**Development of an orally available inhibitor of CLK1 for skipping a mutated dystrophin exon in Duchenne muscular dystrophy**

Yukiya Sako^1^, Kensuke Ninomiya^1^, Yukiko Okuno^2^, Masayasu Toyomoto^1^, Atsushi Nishida^3^, Yuka Koike^1,†^, Kenji Ohe^1^, Isao Kii^1†^, Suguru Yoshida^5^, Naohiro Hashimoto^4^, Takamitsu Hosoya^5^, Masafumi Matsuo^3^, Masatoshi Hagiwara^1*^

*Correspondence: hagiwara.masatoshi.8c@kyoto-u.ac.jp

^1^Department of Anatomy and Developmental Biology, Kyoto University Graduate

School of Medicine, Kyoto, Japan; ^2^Medical Research Support Centre, Kyoto University Graduate School of Medicine, Sakyo-ku, Kyoto 606-8501, Japan; ^3^Department of Medical Rehabilitation, Faculty of Rehabilitation, Kobegakuin University, Kobe, Hyogo, Japan; ^4^Department of Regenerative Medicine, National Centre for Geriatrics and Gerontology, 7-430 Morioka, Oobu, Aichi 474-8522, Japan; ^5^Laboratory of Chemical Bioscience, Institute of Biomaterials and Bioengineering, Tokyo Medical and Dental University, Tokyo, Japan.

^†^Current address: Pathophysiological and Health Science Team, Imaging Application Group, Division of Bio-Function Dynamics Imaging, RIKEN Centre for Life Science Technologies, 6-7-3 Minatojima-minamimachi, Chuo-ku, Kobe, Hyogo 650-0047, Japan.

***Contact information**

Masatoshi Hagiwara, MD and PhD

Department of Anatomy and Developmental Biology, Graduate School of Medicine, Kyoto University, Yoshida-Konoe-cho, Sakyo-ku, Kyoto 606-8501, Japan

E-mail: hagiwara.masatoshi.8c@kyoto-u.ac.jp

Tel: +81-75-753-4341 and Fax: +81-75-751-7529

**Supporting Figures**


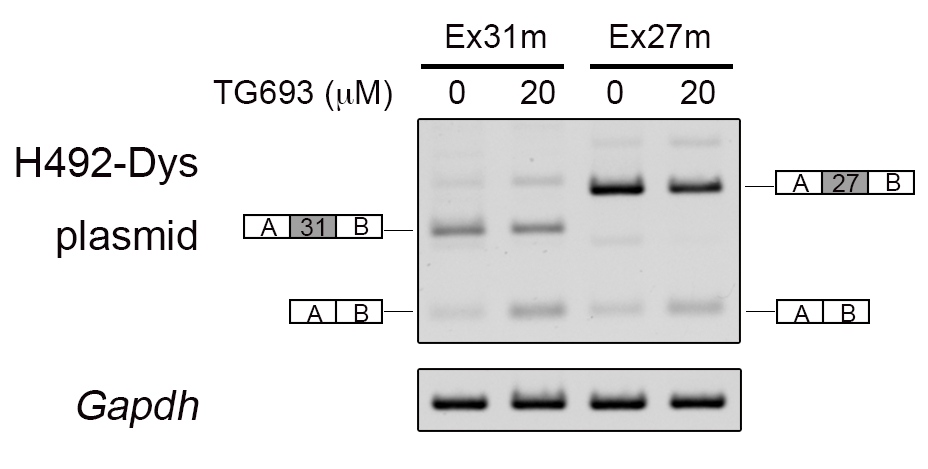


**Supplementary figure S1. TG693 also promotes the skipping of a mutated exon27 in HeLa cells.**

Effect of TG693 on exon 27 skipping was examined using the reporter plasmid. Transfected HeLa cells were incubated in the presence of TG693 or DMSO vehicle for 24 h. Reporter was then analyzed by RT-PCR. *GAPDH* served as a control. Uncroptted images have been provided in Supplementary Fig.S12. Data are representative of three independent experiments.


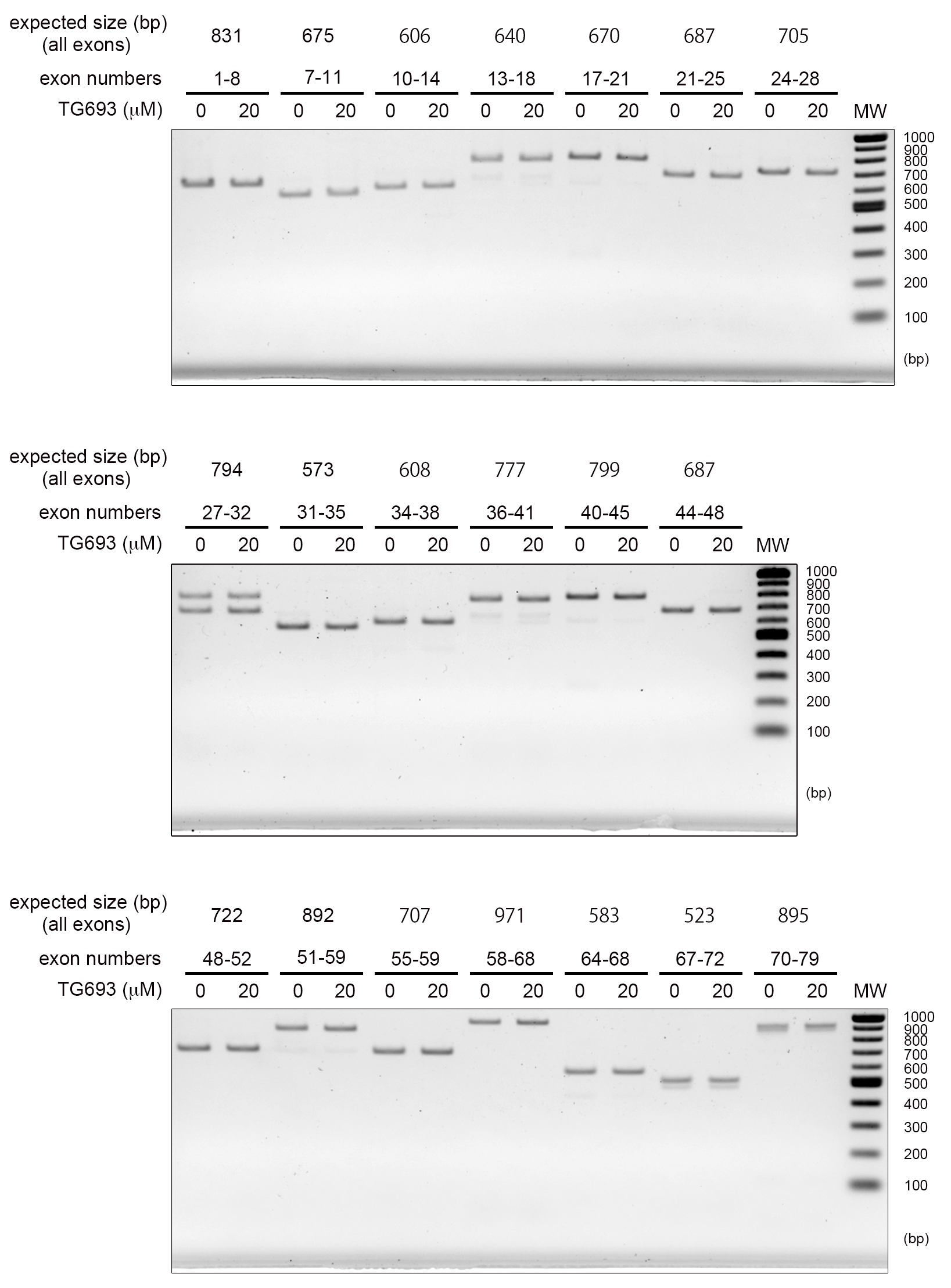


**Supplementary figure S2. TG693 specifically induces skipping of the mutated exon 31 and has almost no effect on splicing of other dystrophin introns in patient-derived cells.**

Immortalized DMD patient-derived cells were treated with 20 μM of TG693 for 2 d. RT-PCR for dystrophin exons. The numbers of exons amplified by specific primer combinations are indicated above the lanes. Uncroptted images have been provided in Supplementary Fig.S13.

**
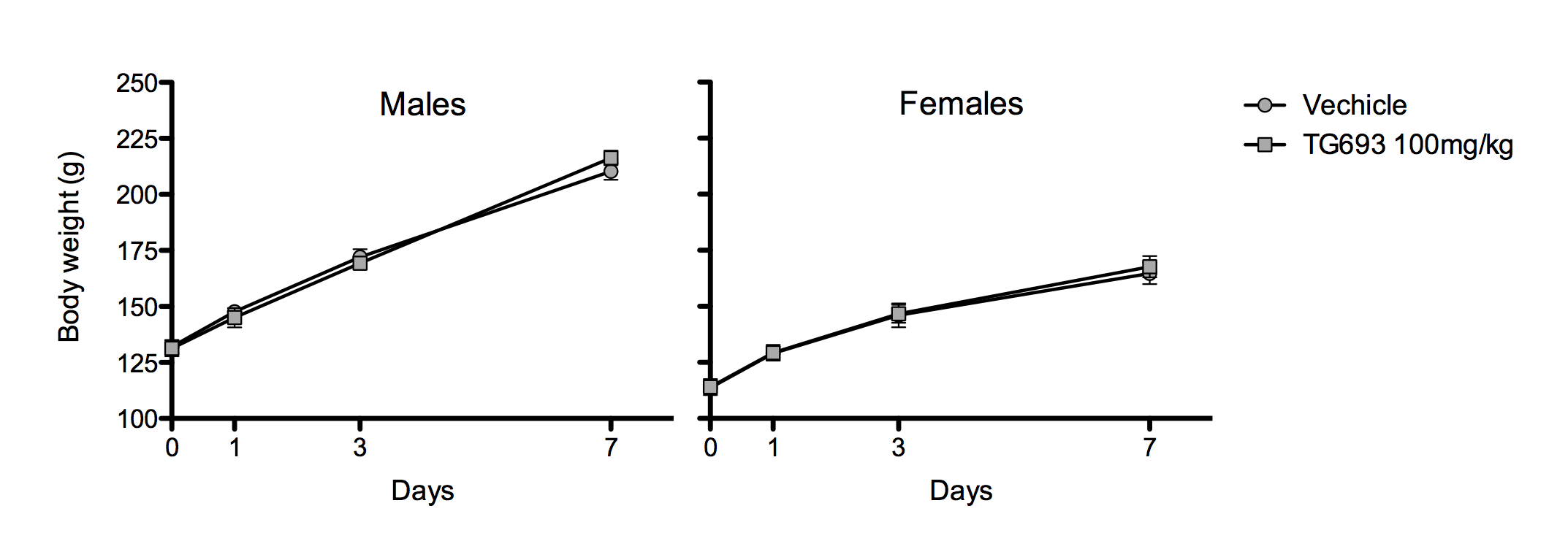
**

**Supplementary figure S3. For seven days single dose oral toxicity studies in rats**

Body weight increased normally in male and female rats administrated orally with TG693 at dose of 100 mg kg^-1^ for 7 days. Control animals were administrated the same amount of vehicle (0.5% methylcellulose) alone. Each value represents the mean and standard deviations (n=3). No mortality and no abnormalities in gross appearance of the animals were observed during the experimental period.


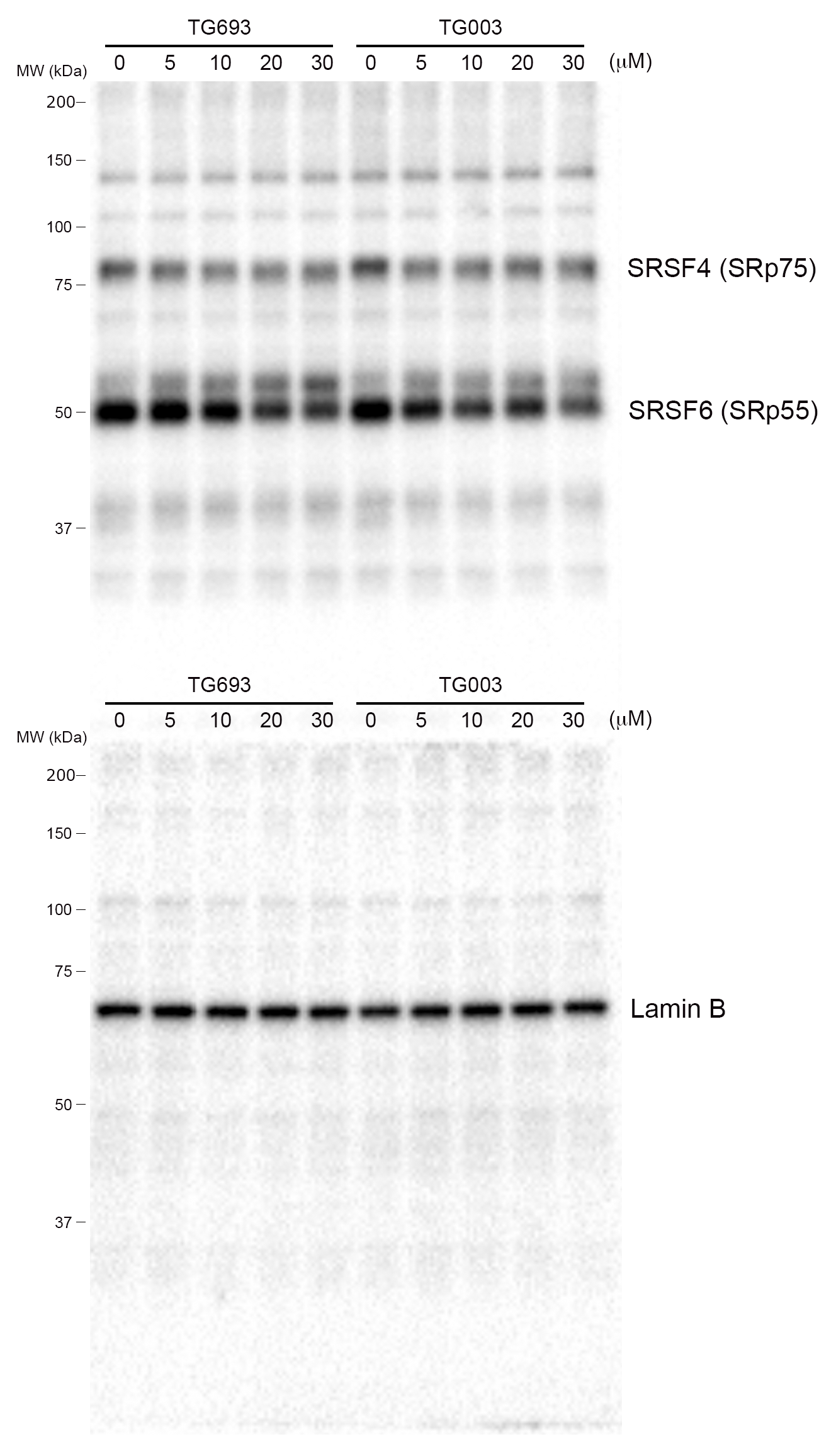


**Supplementary figure S4. Full-length western blot images of Figure 2a**

**
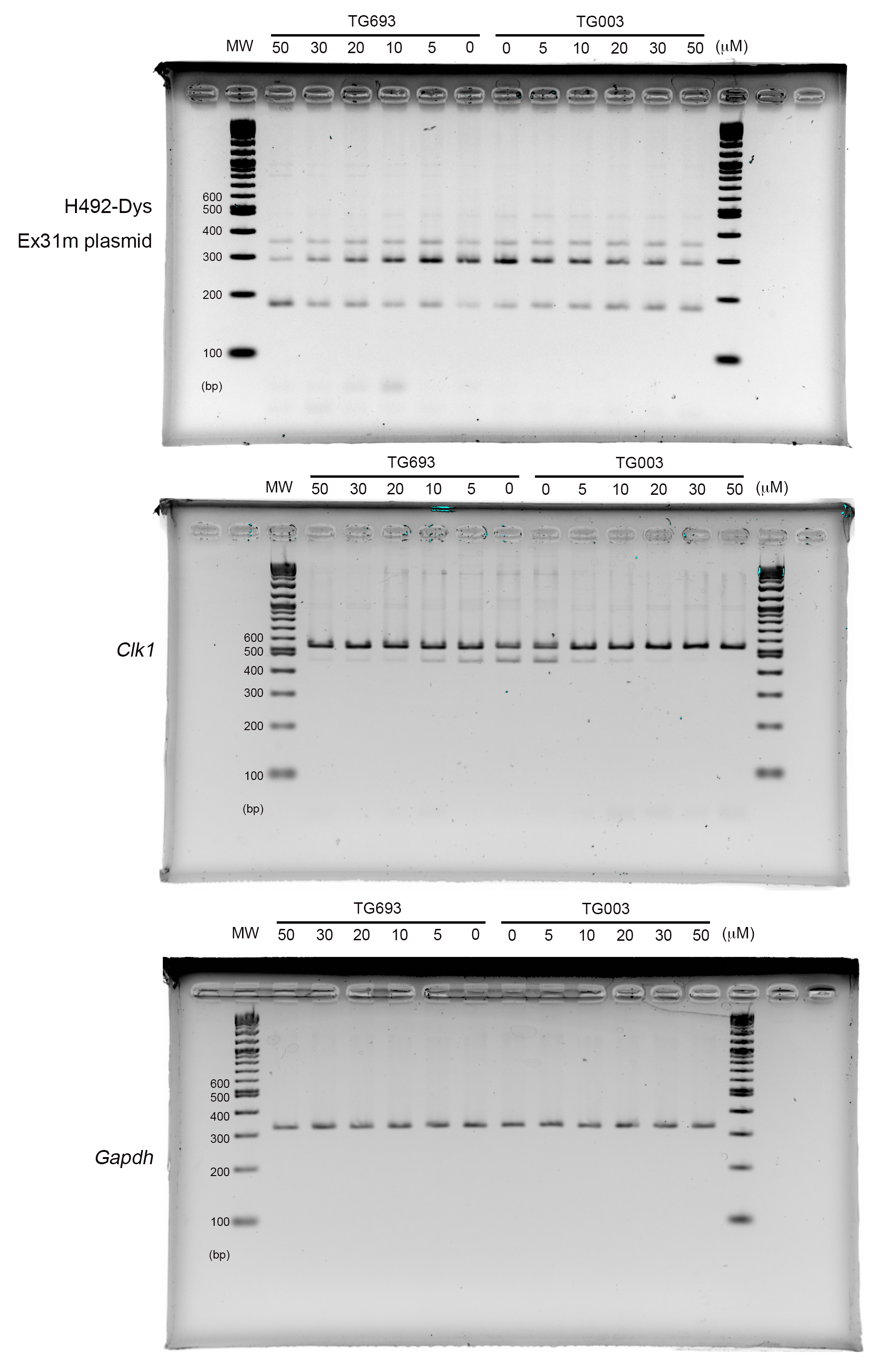
**

**Supplementary figure S5. Agarose gel full images of Figure 2b**


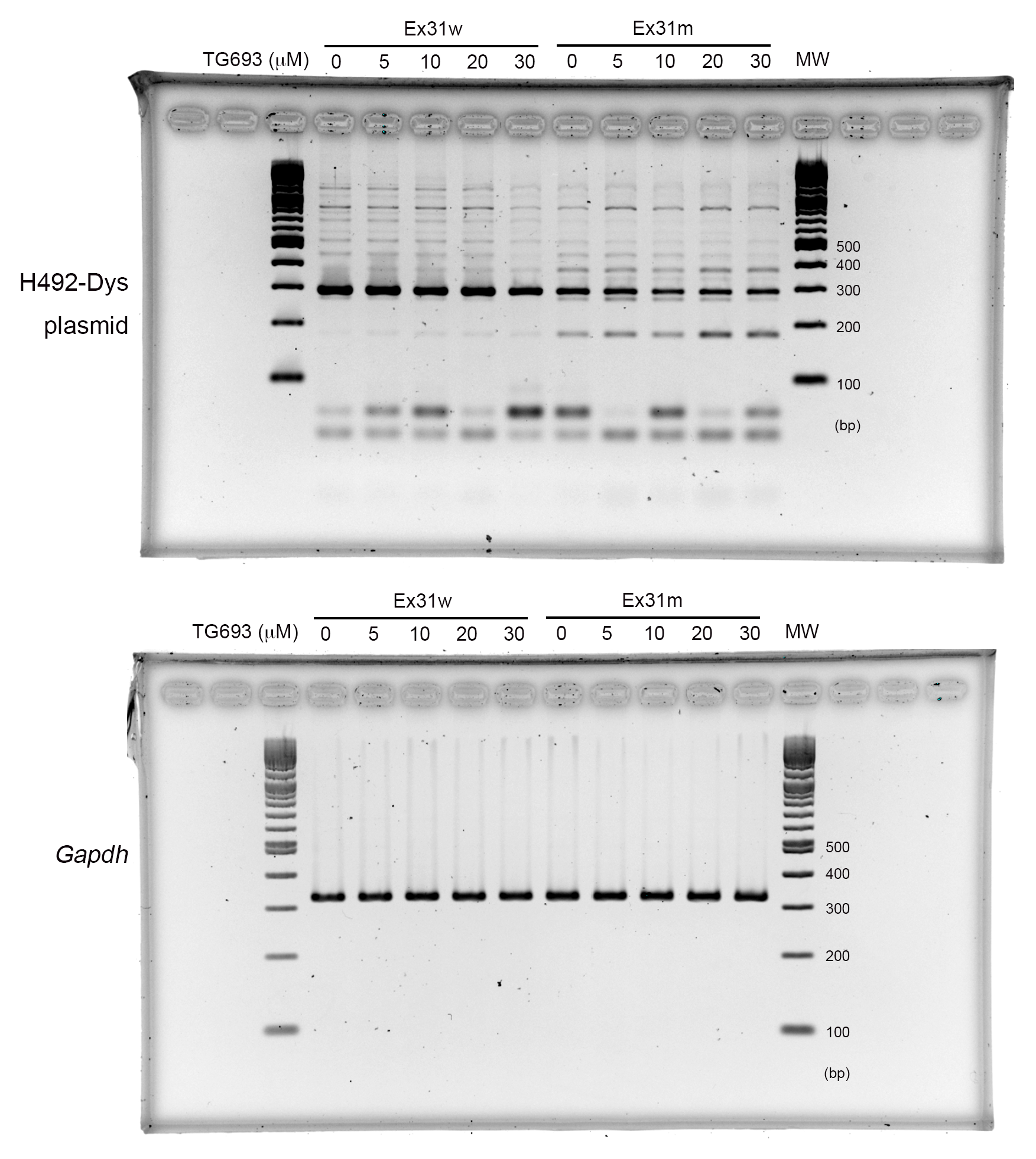


**Supplementary figure S6. Agarose gel full images of Figure 2c**


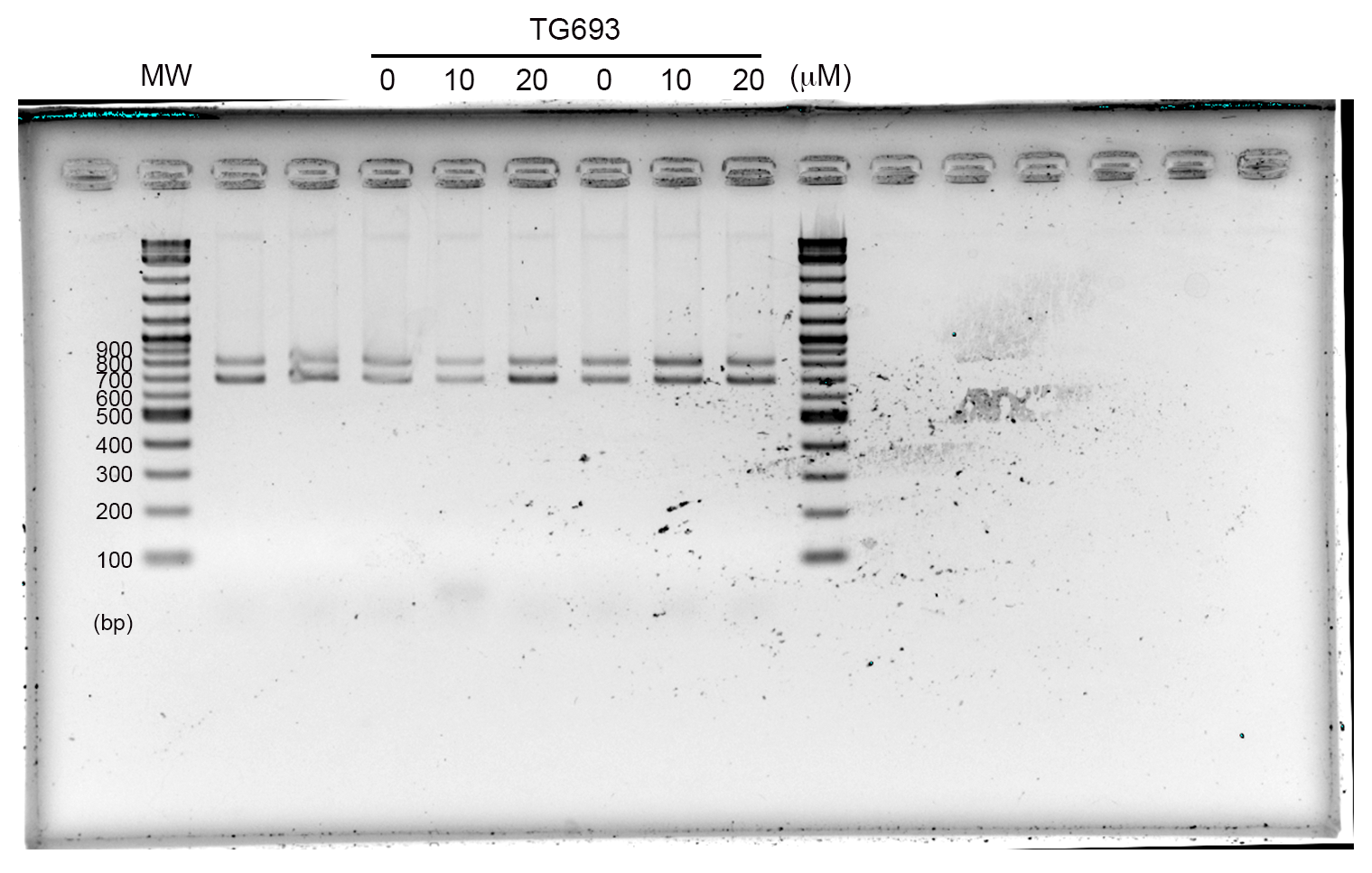


**Supplementary figure S7. An agarose gel full image of Figure 3a**


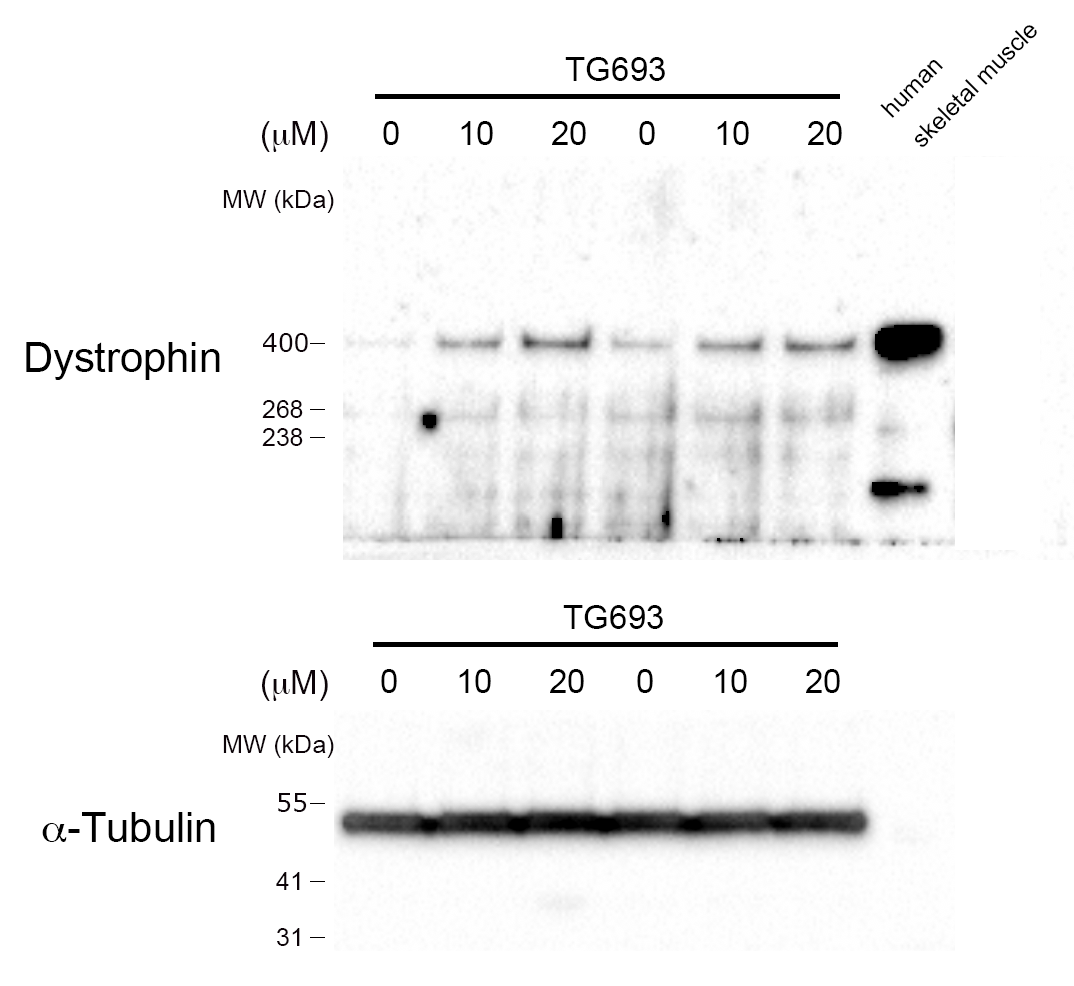


**Supplementary figure S8. Full-length western blot images of Figure 3b**

Skeletal Muscle (Human) Tissue Lysate (Cat.# ab29330, Abcam) was used as a positive control.


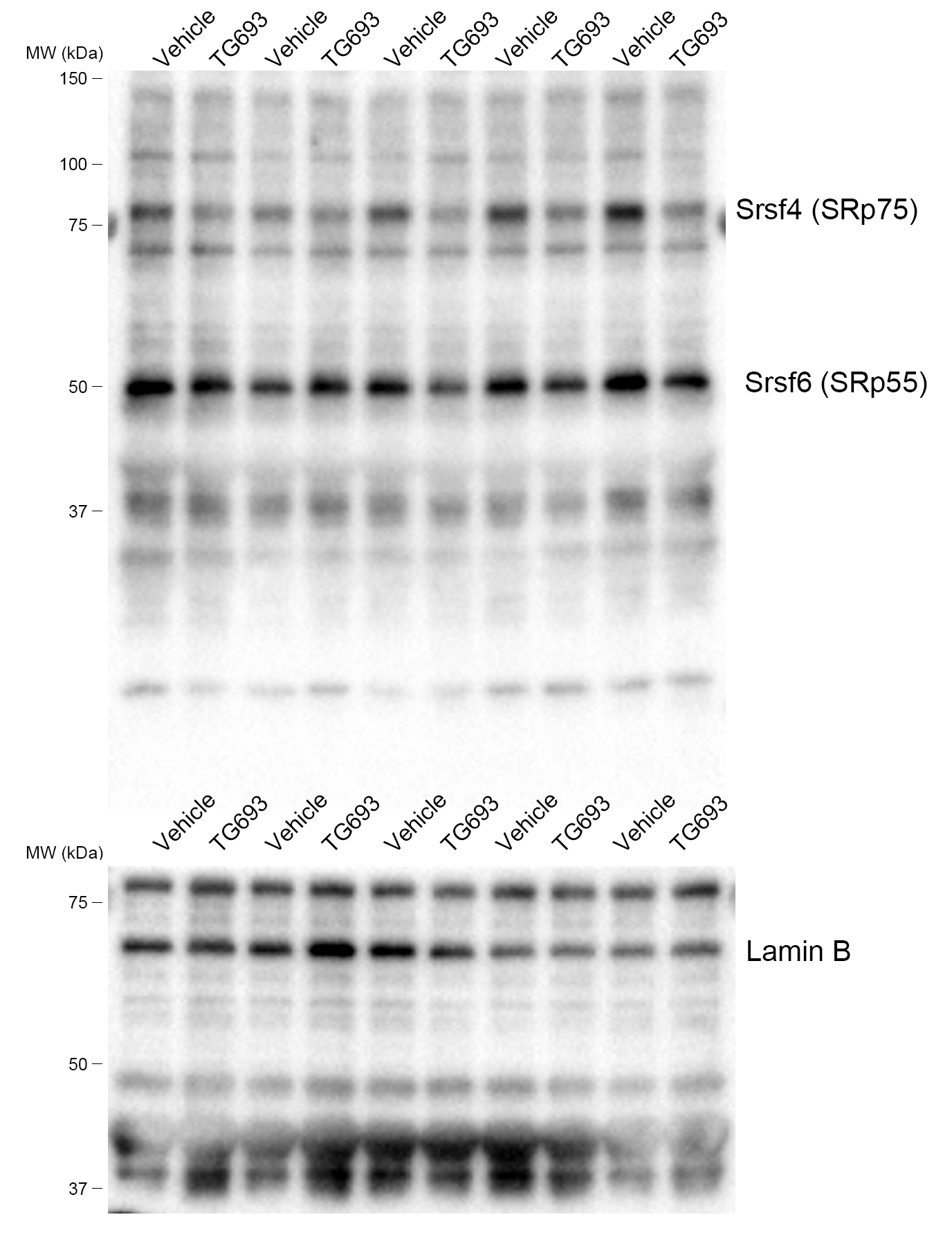


**Supplementary figure S9. Full-length western blot images of Figure 4b**


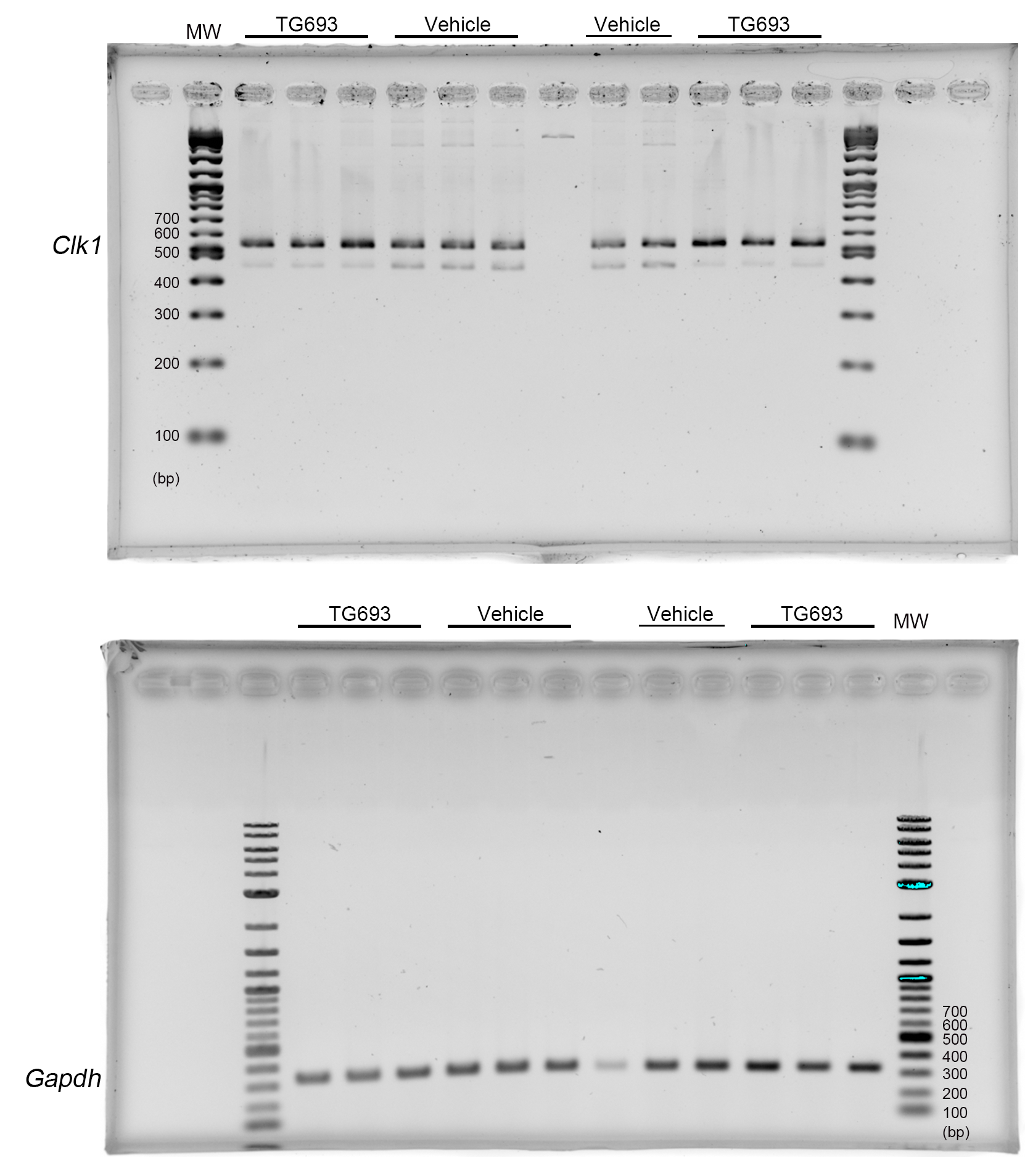


**Supplementary figure S10. Agarose gel full images of Figure 4c**


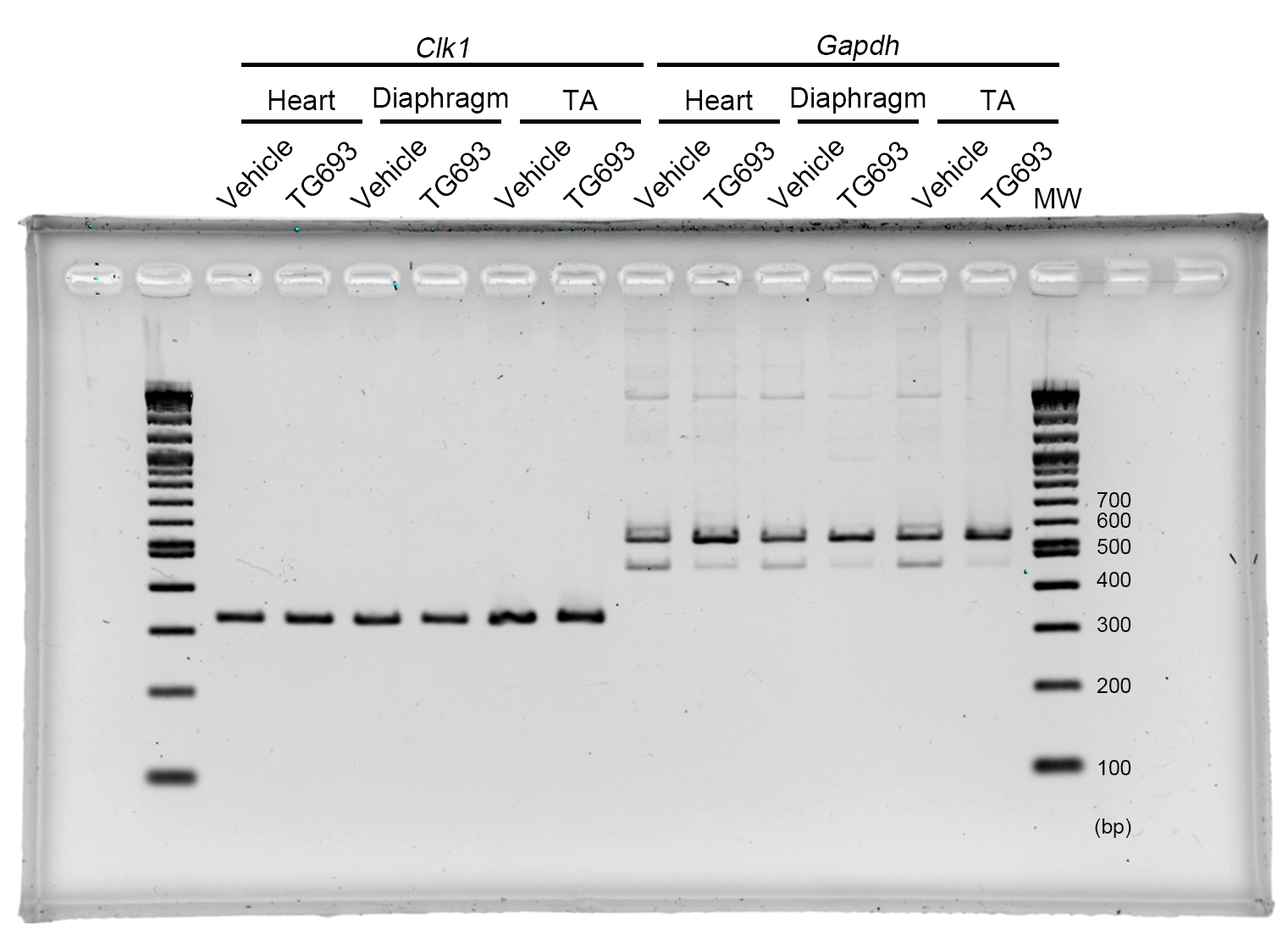


**Supplementary figure S11. An agarose gel full image of Figure 4d**


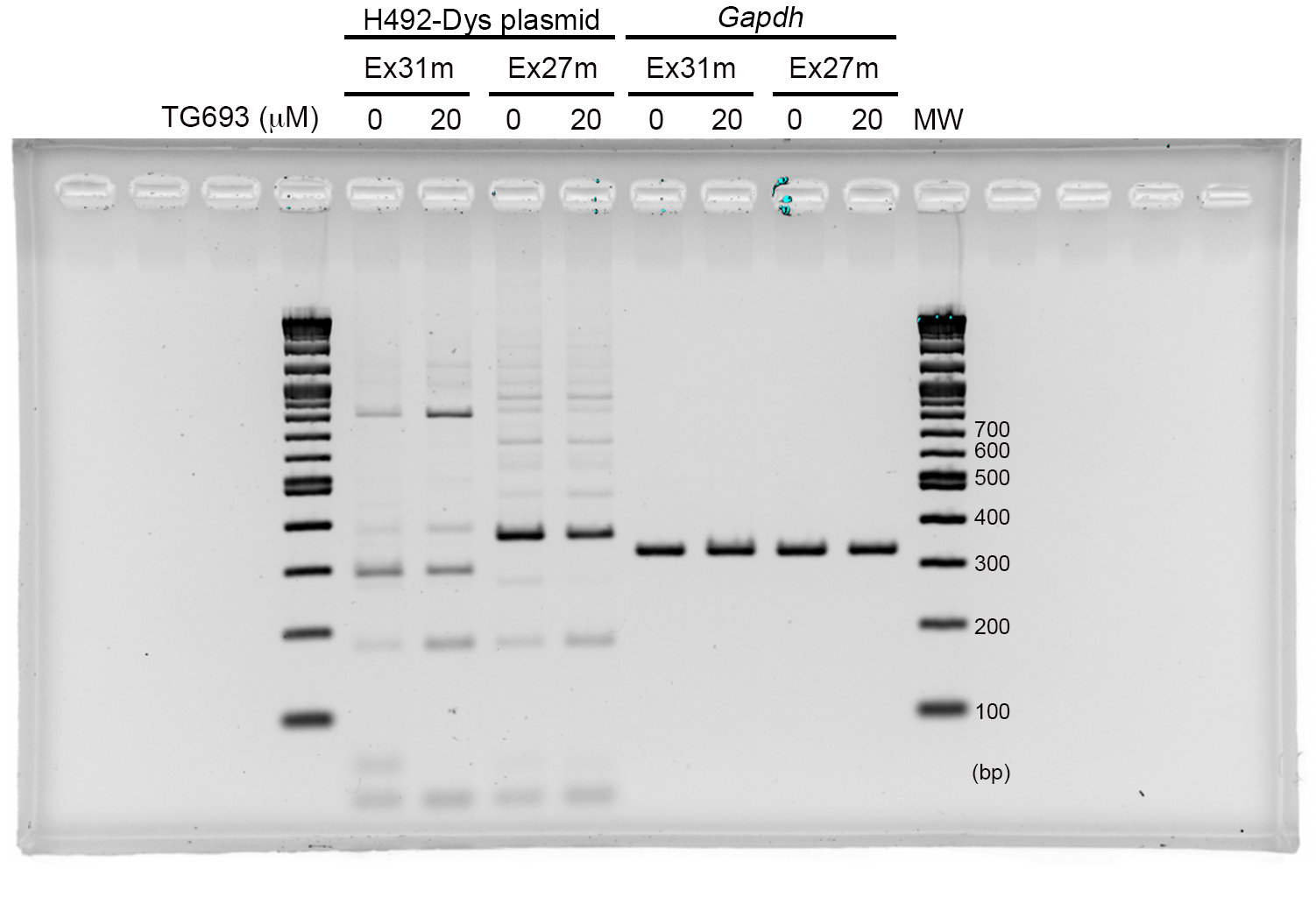


**Supplementary figure S12. An agarose gel full image of Supplementary figure S1**


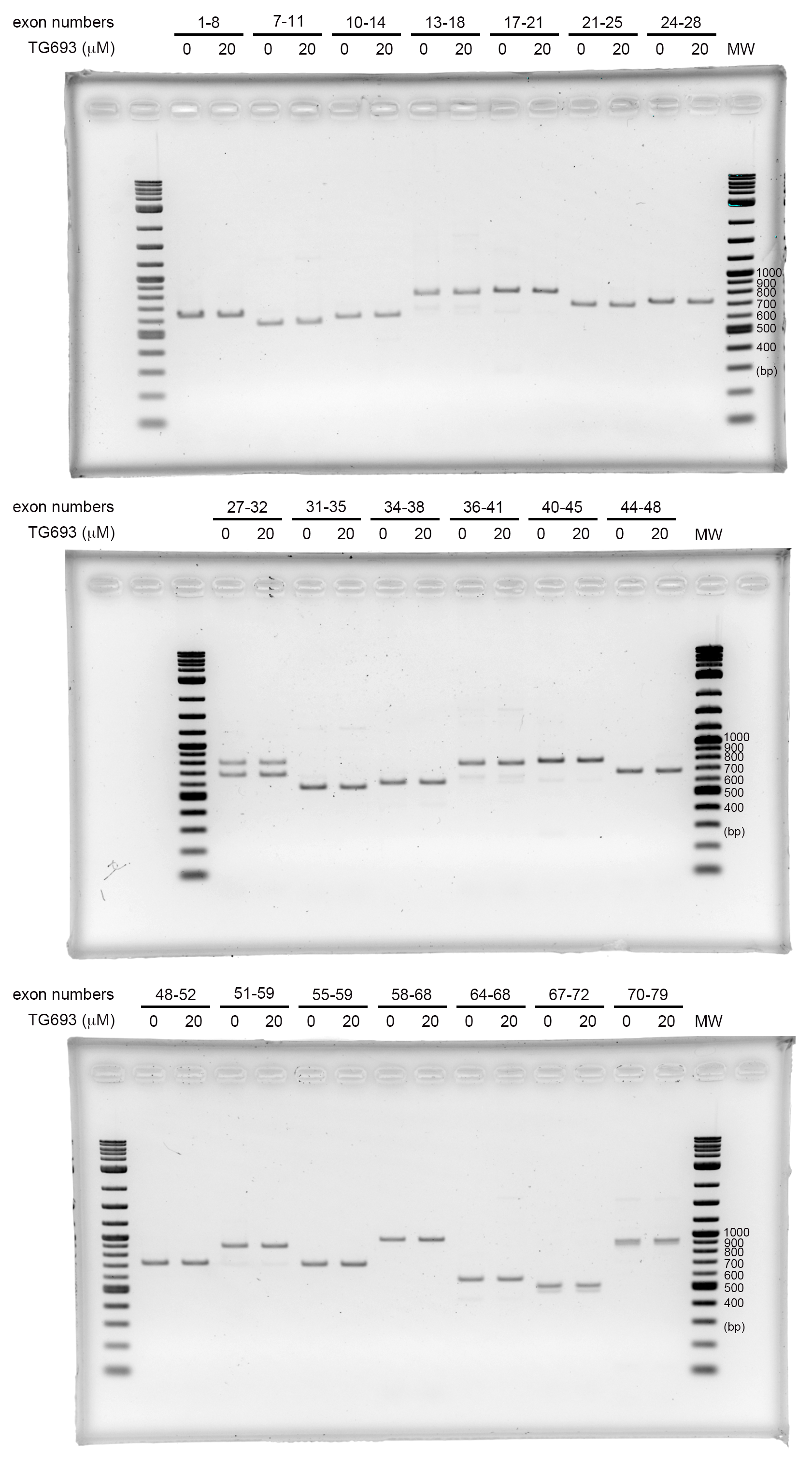


**Supplementary figure S13. Agarose gel full images of Supplementary figure S2**

**Supporting Table**

**Table S1.**

| **TG693 (nM)** | ***K_m_*^apparen^*^t^* (µM)** | ***V_max_*^apparent^ (fmole/min)** | **alpha**^a^ | **alpha'**^b^ |
| --- | --- | --- | --- | --- |
| 250 | 50.17 ± 2.72 | 129.7 ± 1.9 | 3.4 | 1.01 |
| 500 | 88.97 ± 11.3 | 131.1 ± 4.6 | 6.0 | 1.00 |
| 1000 | 142.8 ± 16.13 | 121.8 ± 4.1 | 10.3 | 1.07 |

Kinetic constants (± SEM) were estimated by fitting the data to the Michaelis-Menten equation.

^a^Alpha is calculated by the following equation: alpha = (*K_m_*^apparent^/*V_m_*^apparent^) / (*K_m_*/*V_m_*)

^b^Alpha’ is calculated by the following equation: alpha’ = (1/*V_m_*^apparent^) / (1/*V_m_*)

These values indicated that TG693 ATP-competitively inhibits CLK1.

**Table S2.**

Average of two replicates is shown as percent inhibition of kinase activity in the presence of TG693 (1 μM) relative to solvent control (DMSO).

CLK1 and Haspin were inhibited by over 90%.

| Kinase | %Inhibition | Kinase | %Inhibition |
| --- | --- | --- | --- |
| ABL | -1.3 | ABL(E255K) | -4.2 |
| ABL(T315I) | 6.5 | ACK | -1.1 |
| ALK | 6.1 | ALK(C1156Y) | 0.1 |
| ALK(F1174L) | -6.9 | ALK(G1202R) | 3.8 |
| ALK(L1152insT) | -6.4 | ALK(L1196M) | 2.6 |
| ALK(R1275Q) | -7.6 | EML4-ALK | 2.0 |
| NPM1-ALK | 3.0 | ARG | 4.7 |
| AXL | 3.5 | BLK | -2.2 |
| BMX | 4.6 | BRK | 5.2 |
| BTK | -9.5 | CSK | -1.2 |
| DDR1 | -6.0 | DDR2 | -2.2 |
| EGFR | -10.7 | EGFR(d746-750) | 0.6 |
| EGFR(d746-750/T790M) | 1.5 | EGFR(L858R) | 0.4 |
| EGFR(L861Q) | -5.4 | EGFR(T790M) | -0.2 |
| EGFR(T790M/L858R) | 1.3 | EPHA1 | -2.8 |
| EPHA2 | 5.7 | EPHA3 | 0.4 |
| EPHA4 | 2.4 | EPHA5 | -7.5 |
| EPHA6 | -3.5 | EPHA7 | 5.0 |
| EPHA8 | 7.3 | EPHB1 | 2.5 |
| EPHB2 | 0.3 | EPHB3 | -0.5 |
| EPHB4 | -1.5 | FAK | 3.6 |
| FER | 4.4 | FES | 1.3 |
| FGFR1 | 6.9 | FGFR1(V561M) | -11.6 |
| FGFR2 | 1.7 | FGFR3 | -0.6 |
| FGFR3(K650E) | 5.2 | FGFR3(K650M) | 7.8 |
| FGFR4 | 4.3 | FGFR4(V550E) | -4.1 |
| FGFR4(V550L) | -36.6 | FGR | -5.9 |
| FLT1 | 5.9 | FLT3 | 17.5 |
| FLT4 | -0.3 | FMS | 2.6 |
| FRK | -2.3 | FYN(isoform a) | -0.7 |
| FYN(isoform b) | 0.6 | HCK | -3.0 |
| HER2 | -3.8 | HER4 | 5.8 |
| IGF1R | 3.3 | INSR | -5.9 |
| IRR | 3.0 | ITK | 2.8 |
| JAK1 | -0.7 | JAK2 | 7.9 |
| JAK3 | 11.6 | KDR | 0.1 |
| KIT | -0.9 | KIT(D816E) | -6.8 |
| KIT(D816V) | -1.1 | KIT(D816Y) | -1.7 |
| KIT(T670I) | -0.7 | KIT(V560G) | -2.6 |
| KIT(V654A) | -1.5 | LCK | -8.4 |
| LTK | 3.0 | LYNa | 0.1 |
| LYNb | 0.8 | MER | 3.0 |
| MET | 13.2 | MET(D1228H) | 0.6 |
| MET(M1250T) | -0.8 | MET(Y1235D) | -2.0 |
| MUSK | -5.0 | PDGFRα | 6.2 |
| PDGFRα(D842V) | 0.8 | PDGFRα(T674I) | 7.6 |
| PDGFRα(V561D) | 2.7 | PDGFRβ | 3.0 |
| PYK2 | 3.3 | RET | 4.8 |
| RET(G691S) | 4.9 | RET(M918T) | -2.6 |
| RET(S891A) | -18.0 | RET(Y791F) | 3.0 |
| RON | 1.1 | ROS | 8.1 |
| SRC | 3.2 | SRM | 1.5 |
| SYK | -60.9 | TEC | -0.4 |
| TIE2 | 9.7 | TNK1 | 3.8 |
| TRKA | 5.1 | TRKB | 0.1 |
| TRKC | 7.7 | TXK | -1.0 |
| TYK2 | 0.6 | TYRO3 | 0.3 |
| YES | -9.6 | YES(T348I) | -1.4 |
| AKT1 | -1.3 | AKT2 | -3.0 |
| AKT3 | -4.6 | AMPKα1/β1/γ1 | -6.5 |
| AMPKα2/β1/γ1 | 1.7 | AurA | -6.2 |
| AurA/TPX2 | 8.5 | AurB | 6.1 |
| AurC | 2.5 | BRAF_Cascade | -0.9 |
| BRAF(V600E)_Cascade | 7.9 | BRSK1 | 1.1 |
| BRSK2 | 6.7 | CaMK1α | 1.5 |
| CaMK1δ | 7.0 | CaMK2α | -3.3 |
| CaMK2β | 2.8 | CaMK2γ | -2.4 |
| CaMK2δ | 1.4 | CaMK4 | 0.5 |
| CDC2/CycB1 | 10.3 | CDC7/ASK | 10.8 |
| CDK2/CycA2 | 41.4 | CDK2/CycE1 | 18.1 |
| CDK3/CycE1 | 11.4 | CDK4/CycD3 | 5.4 |
| CDK5/p25 | 21.8 | CDK6/CycD3 | 9.1 |
| CDK7/CycH/MAT1 | 6.2 | CDK9/CycT1 | 57.8 |
| CGK2 | 38.5 | CHK1 | 1.7 |
| CHK2 | -1.0 | CK1α | 11.9 |
| CK1γ1 | 5.0 | CK1γ2 | -3.2 |
| CK1γ3 | -7.7 | CK1δ | 21.3 |
| CK1ε | 8.0 | CK2α1/β | 3.5 |
| CK2α2/β | 14.6 | CLK1 | 92.6 |
| CLK2 | 14.9 | CLK3 | 11.7 |
| COT_Cascade | 1.4 | CRIK | 16.9 |
| DAPK1 | -1.8 | DCAMKL2 | 9.6 |
| DLK_Cascade | 4.0 | DYRK1A | 84.3 |
| DYRK1B | 76.1 | DYRK2 | 77.2 |
| DYRK3 | 64.0 | EEF2K | -15.2 |
| Erk1 | 1.4 | Erk2 | -5.8 |
| Erk5 | 2.2 | GSK3α | 12.6 |
| GSK3β | 8.5 | Haspin | 92.8 |
| HGK | 66.5 | HIPK1 | -3.5 |
| HIPK2 | -2.0 | HIPK3 | -0.7 |
| HIPK4 | 7.6 | IKKα | 14.6 |
| IKKβ | 14.6 | IKKε | 6.1 |
| IRAK1 | 4.2 | IRAK4 | 8.4 |
| JNK1 | -18.0 | JNK2 | -5.9 |
| JNK3 | -4.4 | LATS2 | -3.8 |
| LOK | 5.9 | MAP2K1_Cascade | -7.4 |
| MAP2K2_Cascade | -4.2 | MAP2K3_Cascade | 0.0 |
| MAP2K4_Cascade | -2.8 | MAP2K5_Cascade | 2.4 |
| MAP2K6_Cascade | 3.8 | MAP2K7_Cascade | 0.6 |
| MAP3K1_Cascade | -5.0 | MAP3K2_Cascade | 3.4 |
| MAP3K3_Cascade | 7.5 | MAP3K4_Cascade | -0.7 |
| MAP3K5_Cascade | 3.1 | MAP4K2 | 1.9 |
| MAPKAPK2 | -26.0 | MAPKAPK3 | -13.1 |
| MAPKAPK5 | 2.6 | MARK1 | 13.3 |
| MARK2 | 20.3 | MARK3 | 15.5 |
| MARK4 | 25.1 | MELK | 5.7 |
| MGC42105 | -22.2 | MINK | 23.2 |
| MLK1_Cascade | -1.6 | MLK2_Cascade | -4.5 |
| MLK3_Cascade | -1.6 | MNK1 | 45.7 |
| MNK2 | 57.3 | MOS_Cascade | -5.2 |
| MRCKα | -18.2 | MRCKβ | -7.3 |
| MSK1 | 1.2 | MSK2 | -5.1 |
| MSSK1 | -7.3 | MST1 | -1.9 |
| MST2 | 0.5 | MST3 | 1.0 |
| MST4 | 4.1 | NDR1 | 11.5 |
| NDR2 | 7.5 | NEK1 | -1.5 |
| NEK2 | -11.9 | NEK4 | -4.2 |
| NEK6 | -2.5 | NEK7 | -9.0 |
| NEK9 | -5.1 | NuaK1 | 7.5 |
| NuaK2 | 0.4 | p38α | -0.1 |
| p38β | -1.5 | p38γ | 1.4 |
| p38δ | -0.7 | p70S6K | 46.0 |
| p70S6Kβ | 18.7 | PAK1 | -3.8 |
| PAK2 | -51.3 | PAK4 | -13.7 |
| PAK5 | -14.0 | PAK6 | -4.3 |
| PASK | 14.7 | PBK | -3.5 |
| PDHK2 | -0.8 | PDHK4 | 3.1 |
| PDK1 | -3.9 | PEK | 5.0 |
| PGK | 11.0 | PHKG1 | 0.0 |
| PHKG2 | 6.3 | PIM1 | 1.2 |
| PIM2 | -3.5 | PIM3 | 6.6 |
| PKACα | 9.5 | PKACβ | 3.1 |
| PKACγ | 1.5 | PKCα | -5.4 |
| PKCβ1 | -11.4 | PKCβ2 | -11.8 |
| PKCγ | -11.4 | PKCδ | 1.5 |
| PKCε | 12.3 | PKCζ | -9.7 |
| PKCη | 5.8 | PKCθ | -12.6 |
| PKCι | -6.8 | PKD1 | -15.8 |
| PKD2 | 5.4 | PKD3 | -9.0 |
| PKN1 | 34.5 | PKR | 1.2 |
| PLK1 | -8.5 | PLK2 | 2.9 |
| PLK3 | 1.7 | PRKX | 12.2 |
| QIK | 3.4 | RAF1_Cascade | -5.1 |
| ROCK1 | 27.6 | ROCK2 | 46.3 |
| RSK1 | -4.7 | RSK2 | 2.1 |
| RSK3 | -4.8 | RSK4 | -6.5 |
| SGK | 5.6 | SGK2 | -5.4 |
| SGK3 | -3.2 | SIK | 3.2 |
| skMLCK | 0.9 | SLK | 1.2 |
| SRPK1 | 0.6 | SRPK2 | 0.5 |
| TAK1-TAB1_Cascade | 2.9 | TAOK2 | 2.0 |
| TBK1 | 5.8 | TNIK | 48.6 |
| TSSK1 | 3.0 | TSSK2 | -0.1 |
| TSSK3 | 0.8 | WNK1 | 4.1 |
| WNK2 | 4.0 | WNK3 | -4.9 |
| PIK3CA/PIK3R1 | 36.8 | SPHK1 | -2.3 |
| SPHK2 | 4.1 |  | |

**Table S3.**

The commercially available antibodies used in this study.

| Antibody |  | Clone ID | Vendor | Cat. No. |
| --- | --- | --- | --- | --- |
| SR proteins | rmouse monoclonal | 1H4 | Thermo Fisher Scientific | 33-9400 |
| α-Tubullin | rmouse monoclonal | DM1A | Thermo Fisher Scientific | MS-581-P1 |
| Dystrophin | rabbit polyclonal |  | Abcam | ab15277 |
| Lamin B | goat polyclonal | M-20 | Santa Cruz Biotechnology | sc-6217 |

**Table S4.**

Primers used to investigate splicing of all dystrophin introns in patient-derived cells (Supplementary figure S3)

| Exons amplified | Location | Sequence |
| --- | --- | --- |
| 1-8 | 1-22 | ATGCTTTGGTGGGAAGAAGTAG |
| 1-8 | 831-809 | CTGTTGAGAATAGTGCATTTGAT |
| 7-11 | 556-579 | GTGGTTTGCCAGCAGTCAGCCACA |
| 7-11 | 1230-108 | TCCTGTTCCAATCAGCTTACTTC |
| 10-14 | 1084-1104 | TTGCAAGCACAAGGAGAGATT |
| 10-14 | 1689-1669 | ACGTTGCCATTTGAGAAGGAT |
| 13-18 | 1579-1599 | GCTGCTTTGGAAGAACAACTT |
| 13-18 | 2218-2197 | CTTCTGAGCGAGTAATCCAGCT |
| 17-21 | 2134-2156 | AGGCAGATTACTGTGGATTCTGA |
| 17-21 | 2803-2783 | TTGTCTGTAGCTCTTTCTCTC |
| 21-25 | 2650-2669 | CAACCTCAAATTGAACGATT |
| 21-25 | 3336-3316 | CCCACCTTCATTGACACTGTT |
| 24-28 | 3112-3134 | GAGCATTGTCAAAAGCTAGAGGA |
| 24-28 | 3816-3793 | CAATAACTCATGCCAACATGCCCA |
| 27-32 | 3688-3708 | CCTGTAGCACAAGAGGCCTTA |
| 27-32 | 4481-4460 | TCCACACTCTTTGTTTCCAATG |
| 31-35 | 4309-4328 | GCCCAAAGAGTCCTGTCTCA |
| 31-35 | 4881-4862 | GTGCACCTTCTGTTTCTCAA |
| 34-38 | 4753-4772 | GAATGGCTGGCAGCTACAGA |
| 34-38 | 5360-5338 | TTAAACTGCTCCAATTCCTTCAA |
| 36-41 | 5050-5069 | TTTGACCAGAATGTGGACCA |
| 36-41 | 5826-5806 | TGCGGCCCCATCCTCAGACAA |
| 40-45 | 5704-5725 | AGCCTACCTGAGCCCAGAGATG |
| 40-45 | 6502-6483 | CTTCCCCAGTTGCATTCAAT |
| 44-48 | 6367-6393 | GCTGAACAGTTTCTCAGAAAGACACAA |
| 44-48 | 7053-7033 | CAACTGATTCCTAATAGGAGA |
| 48-52 | 6937-6957 | CAAGGAGAAATTGAAGCTCAA |
| 48-52 | 7658-7636 | CGATCCGTAATGATTGTTCTAGC |
| 51-59 | 7435-7455 | TGGACAGAACTTACCGACTGG |
| 51-59 | 8326-8307 | GTAACAGGACTGCATCATCG |
| 55-59 | 8040-8059 | AGAGGCTGCTTTGGAAGAAA |
| 55-59 | 8746-8725 | CCCACTCAGTATTGACCTCCTC |
| 58-68 | 8619-8638 | GACAGAGCAGCCTTTGGAAG |
| 58-68 | 9589-9568 | GGACACGGATCCTCCCTGTTCG |
| 64-68 | 9334-9353 | CTCCGAAGACTGCAGAAGGC |
| 64-68 | 9916-9898 | TTTCTGCAGCAGCCACTCT |
| 67-72 | 9775-9792 | ATTGAGCCAAGTGTCCGG |
| 67-72 | 10297-10277 | TATCATCGTGTGAAAGCTGAG |
| 70-79 | 10107-10127 | GAATGGGCTACCTGCCAGTG |
| 70-79 | 11162-11142 | ATCGCTCTGCCCAAATCATCTG |

**Table S5.**

Reaction conditions for each kinase (Figure 1e and Supplementary Table S1)

| **Kinase** | **Platform** | **Substrate** | | **ATP (µM)** | | **Metal** | |
| --- | --- | --- | --- | --- | --- | --- | --- |
|  |  | **Name** | **(nM)** | **Km** | **Assay** | **Name** | **(mM)** |
| ABL | MSA | ABLtide | 1000 | 16 | 25 | Mg | 5 |
| ABL[E255K] | MSA | ABLtide | 1000 | 17 | 25 | Mg | 5 |
| ABL[T315I] | MSA | ABLtide | 1000 | 4 | 5 | Mg | 5 |
| ACK ^1)^ | MSA | WASP peptide | 1000 | 97 | 100 | Mg | 5 |
| ALK | MSA | Srctide | 1000 | 57 | 50 | Mg | 5 |
| ALK[C1156Y] | MSA | Srctide | 1000 | 64 | 75 | Mg | 5 |
| ALK[F1174L] | MSA | Srctide | 1000 | 49 | 50 | Mg | 5 |
| ALK[G1202R] | MSA | Srctide | 1000 | 31 | 50 | Mg | 5 |
| ALK[L1152insT] | MSA | Srctide | 1000 | 108 | 100 | Mg | 5 |
| ALK[L1196M] | MSA | Srctide | 1000 | 57 | 75 | Mg | 5 |
| ALK[R1275Q] | MSA | Srctide | 1000 | 84 | 100 | Mg | 5 |
| EML4-ALK ^1)^ | MSA | Srctide | 1000 | 43 | 50 | Mg | 5 |
| NPM1-ALK | MSA | Srctide | 1000 | 57 | 50 | Mg | 5 |
| ARG | MSA | ABLtide | 1000 | 24 | 25 | Mg | 5 |
| AXL | MSA | CSKtide | 1000 | 32 | 50 | Mg | 5 |
| BLK | MSA | Srctide | 1000 | 62 | 75 | Mg | 5 |
| BMX | MSA | Srctide | 1000 | 75 | 75 | Mg | 5 |
| BRK ^1)^ | MSA | Blk/Lyntide | 1000 | 250 | 250 | Mg | 5 |
| BTK | MSA | Srctide | 1000 | 72 | 75 | Mg | 5 |
| CSK ^1)^ | MSA | Srctide | 1000 | 4.8 | 5 | Mg+Mn | 5+1 |
| DDR1 ^1)^ | MSA | IRS1 | 1000 | 94 | 100 | Mg | 5 |
| DDR2 ^1)^ | MSA | IRS1 | 1000 | 38 | 50 | Mg | 5 |
| EGFR | MSA | Srctide | 1000 | 2.7 | 5 | Mg+Mn | 5+1 |
| EGFR[d746-750] | MSA | Srctide | 1000 | 19 | 25 | Mg+Mn | 5+1 |
| EGFR[d746-750/ T790M] | MSA | Srctide | 1000 | 5.4 | 5 | Mg+Mn | 5+1 |
| EGFR[L858R] | MSA | Srctide | 1000 | 9.8 | 10 | Mg+Mn | 5+1 |
| EGFR[L861Q] | MSA | Srctide | 1000 | 7.5 | 10 | Mg+Mn | 5+1 |
| EGFR[T790M] | MSA | Srctide | 1000 | 0.9 | 1 | Mg+Mn | 5+1 |
| EGFR[T790M/L858R] | MSA | Srctide | 1000 | 1.9 | 2 | Mg+Mn | 5+1 |
| EPHA1 | MSA | Blk/Lyntide | 1000 | 22 | 25 | Mg | 5 |
| EPHA2 | MSA | Blk/Lyntide | 1000 | 67 | 75 | Mg | 5 |
| EPHA3 | MSA | Blk/Lyntide | 1000 | 170 | 150 | Mg | 5 |
| EPHA4 | MSA | Blk/Lyntide | 1000 | 52 | 50 | Mg | 5 |
| EPHA5 | MSA | Blk/Lyntide | 1000 | 56 | 50 | Mg | 5 |
| EPHA6 | MSA | Blk/Lyntide | 1000 | 27 | 25 | Mg | 5 |
| EPHA7 | MSA | Blk/Lyntide | 1000 | 58 | 50 | Mg | 5 |
| EPHA8 | MSA | Blk/Lyntide | 1000 | 69 | 75 | Mg | 5 |
| EPHB1 | MSA | Blk/Lyntide | 1000 | 29 | 25 | Mg | 5 |
| EPHB2 | MSA | Blk/Lyntide | 1000 | 86 | 100 | Mg | 5 |
| EPHB3 | MSA | Blk/Lyntide | 1000 | 49 | 50 | Mg | 5 |
| EPHB4 | MSA | Blk/Lyntide | 1000 | 56 | 50 | Mg | 5 |
| FAK ^1)^ | MSA | Blk/Lyntide | 1000 | 25 | 25 | Mg | 5 |
| FER | MSA | Srctide | 1000 | 26 | 25 | Mg | 5 |
| FES | MSA | Srctide | 1000 | 43 | 50 | Mg | 5 |
| FGFR1 | MSA | CSKtide | 1000 | 89 | 100 | Mg | 5 |
| FGFR1[V561M] | MSA | CSKtide | 1000 | 33 | 50 | Mg | 5 |
| FGFR2 | MSA | CSKtide | 1000 | 66 | 75 | Mg | 5 |
| FGFR3 | MSA | CSKtide | 1000 | 43 | 50 | Mg | 5 |
| FGFR3[K650E] | MSA | CSKtide | 1000 | 41 | 50 | Mg | 5 |
| FGFR3[K650M] | MSA | CSKtide | 1000 | 17 | 25 | Mg | 5 |
| FGFR4 | MSA | CSKtide | 1000 | 230 | 250 | Mg | 5 |
| FGFR4[V550E] | MSA | CSKtide | 1000 | 210 | 200 | Mg | 5 |
| FGFR4[V550L] | MSA | CSKtide | 1000 | 160 | 150 | Mg | 5 |
| FGR | MSA | Srctide | 1000 | 34 | 50 | Mg | 5 |
| FLT1 | MSA | CSKtide | 1000 | 140 | 150 | Mg | 5 |
| FLT3 | MSA | Srctide | 1000 | 94 | 100 | Mg | 5 |
| FLT4 | MSA | CSKtide | 1000 | 72 | 75 | Mg | 5 |
| FMS | MSA | Srctide | 1000 | 26 | 25 | Mg | 5 |
| FRK | MSA | Srctide | 1000 | 62 | 75 | Mg | 5 |
| FYN[isoform a] | MSA | Srctide | 1000 | 36 | 50 | Mg | 5 |
| FYN[isoform b] | MSA | Srctide | 1000 | 20 | 25 | Mg | 5 |
| HCK | MSA | Srctide | 1000 | 11 | 10 | Mg | 5 |
| HER2 | MSA | Srctide | 1000 | 9.4 | 10 | Mn | 5 |
| HER4 | MSA | Srctide | 1000 | 27 | 25 | Mg | 5 |
| IGF1R | MSA | IRS1 | 1000 | 63 | 75 | Mg | 5 |
| INSR | MSA | IRS1 | 1000 | 58 | 50 | Mg | 5 |
| IRR | MSA | IRS1 | 1000 | 64 | 75 | Mg | 5 |
| ITK | MSA | Srctide | 1000 | 6.1 | 10 | Mg | 5 |
| JAK1 ^1)5)^ | MSA | JAK1 substrate peptide | 1000 | 68 | 75 | Mg | 5 |
| JAK2 | MSA | Srctide | 1000 | 13 | 10 | Mg | 5 |
| JAK3 | MSA | Srctide | 1000 | 3.5 | 5 | Mg | 5 |
| KDR | MSA | CSKtide | 1000 | 74 | 75 | Mg | 5 |
| KIT ^5)^ | MSA | Srctide | 1000 | 370 | 400 | Mg | 5 |
| KIT[D816E] ^5)^ | MSA | Srctide | 1000 | 40 | 50 | Mg | 5 |
| KIT[D816V] ^5)^ | MSA | Srctide | 1000 | 14 | 10 | Mg | 5 |
| KIT[D816Y] ^5)^ | MSA | Srctide | 1000 | 22 | 25 | Mg | 5 |
| KIT[T670I] ^5)^ | MSA | Srctide | 1000 | 100 | 100 | Mg | 5 |
| KIT[V560G] ^5)^ | MSA | Srctide | 1000 | 110 | 250 | Mg | 5 |
| KIT[V654A] ^5)^ | MSA | Srctide | 1000 | 220 | 250 | Mg | 5 |
| LCK | MSA | Srctide | 1000 | 14 | 10 | Mg | 5 |
| LTK | MSA | Srctide | 1000 | 49 | 50 | Mg | 5 |
| LYNa | MSA | Srctide | 1000 | 14 | 10 | Mg | 5 |
| LYNb | MSA | Srctide | 1000 | 18 | 25 | Mg | 5 |
| MER | MSA | CSKtide | 1000 | 36 | 50 | Mg | 5 |
| MET | MSA | Srctide | 1000 | 27 | 25 | Mg | 5 |
| MET[D1228H] | MSA | Srctide | 1000 | 25 | 25 | Mg | 5 |
| MET[M1250T] | MSA | Srctide | 1000 | 17 | 25 | Mg | 5 |
| MET[Y1235D] | MSA | Srctide | 1000 | 71 | 75 | Mg | 5 |
| MUSK ^1)^ | MSA | CSKtide | 1000 | 14 | 10 | Mg+Mn | 5+1 |
| PDGFRα | MSA | CSKtide | 1000 | 28 | 25 | Mg | 5 |
| PDGFRα [D842V] | MSA | CSKtide | 1000 | 21 | 25 | Mg | 5 |
| PDGFRα [T674I] ^1)^ | MSA | CSKtide | 1000 | 11 | 10 | Mg | 5 |
| PDGFRα [V561D] | MSA | CSKtide | 1000 | 35 | 50 | Mg | 5 |
| PDGFRβ | MSA | CSKtide | 1000 | 23 | 25 | Mg | 5 |
| PYK2 | MSA | Blk/Lyntide | 1000 | 56 | 50 | Mg | 5 |
| RET | MSA | CSKtide | 1000 | 7.5 | 10 | Mg | 5 |
| RET[G691S] | MSA | CSKtide | 1000 | 13 | 10 | Mg | 5 |
| RET[M918T] | MSA | CSKtide | 1000 | 4.2 | 5 | Mg | 5 |
| RET[S891A] | MSA | CSKtide | 1000 | 11 | 10 | Mg | 5 |
| RET[Y791F] | MSA | CSKtide | 1000 | 29 | 25 | Mg | 5 |
| RON | MSA | Srctide | 1000 | 27 | 25 | Mg | 5 |
| ROS | MSA | IRS1 | 1000 | 37 | 50 | Mg | 5 |
| SRC | MSA | Srctide | 1000 | 31 | 50 | Mg | 5 |
| SRM | MSA | Blk/Lyntide | 1000 | 38 | 50 | Mg | 5 |
| SYK | MSA | Blk/Lyntide | 1000 | 26 | 25 | Mg | 5 |
| TEC | MSA | Srctide | 1000 | 55 | 50 | Mg | 5 |
| TIE2 | MSA | Blk/Lyntide | 1000 | 94 | 100 | Mg | 5 |
| TNK1 ^1)^ | MSA | CSKtide | 1000 | 71 | 75 | Mg | 5 |
| TRKA | MSA | CSKtide | 1000 | 65 | 75 | Mg | 5 |
| TRKB | MSA | Srctide | 1000 | 80 | 75 | Mg | 5 |
| TRKC | MSA | Srctide | 1000 | 47 | 50 | Mg | 5 |
| TXK ^1)^ | MSA | Srctide | 1000 | 110 | 100 | Mg | 5 |
| TYK2 ^1)^ | MSA | Srctide | 1000 | 18 | 25 | Mg | 5 |
| TYRO3 | MSA | CSKtide | 1000 | 80 | 75 | Mg | 5 |
| YES | MSA | Srctide | 1000 | 13 | 10 | Mg | 5 |
| YES[T348I] | MSA | Srctide | 1000 | 8.5 | 10 | Mg | 5 |
| AKT1 | MSA | Crosstide | 1000 | 31 | 50 | Mg | 5 |
| AKT2 | MSA | Crosstide | 1000 | 110 | 100 | Mg | 5 |
| AKT3 | MSA | Crosstide | 1000 | 54 | 50 | Mg | 5 |
| AMPKα1/β1/γ1 | MSA | SAMS peptide | 1000 | 130 | 150 | Mg | 5 |
| AMPKα2/β1/γ1 | MSA | SAMS peptide | 1000 | 100 | 100 | Mg | 5 |
| AurA | MSA | Kemptide | 1000 | 27 | 25 | Mg | 5 |
| AurA/TPX2 ^9)^ | MSA | Kemptide | 1000 | 1.7 | 2 | Mg | 5 |
| AurB/INCENP | MSA | Kemptide | 1000 | 16 | 25 | Mg | 5 |
| AurC | MSA | Kemptide | 1000 | 24 | 25 | Mg | 5 |
| BRAF_Cascade | MSA | MAP2K1 | 1 | - | 1000 | Mg | 5 |
|  |  | Erk2 | 2.5 |  |  |  |  |
|  |  | Modified Erktide | 1000 |  |  |  |  |
| BRAF[V600E]_Cascade | MSA | MAP2K1 | 1 | - | 1000 | Mg | 5 |
|  |  | Erk2 | 2.5 |  |  |  |  |
|  |  | Modified Erktide | 1000 |  |  |  |  |
| BRSK1 | MSA | CHKtide | 1000 | 30 | 25 | Mg | 5 |
| BRSK2 | MSA | CHKtide | 1000 | 31 | 50 | Mg | 5 |
| CaMK1α ^1)2)^ | MSA | GS peptide | 1000 | 750 | 1000 | Mg | 5 |
| CaMK1δ ^1)2)^ | MSA | Synapsin peptide | 1000 | 11 | 10 | Mg | 5 |
| CaMK2α ^2)^ | MSA | GS peptide | 1000 | 33 | 50 | Mg | 5 |
| CaMK2β ^2)^ | MSA | GS peptide | 1000 | 19 | 25 | Mg | 5 |
| CaMK2γ ^2)^ | MSA | GS peptide | 1000 | 23 | 25 | Mg | 5 |
| CaMK2δ ^2)^ | MSA | GS peptide | 1000 | 6.3 | 5 | Mg | 5 |
| CaMK4 ^2)^ | MSA | GS peptide | 1000 | 20 | 25 | Mg | 5 |
| CDC2/CycB1 | MSA | Modified Histone H1 | 1000 | 34 | 50 | Mg | 5 |
| CDC7/ASK ^1)^ | MSA | MCM2 peptide | 1000 | 2.8 | 5 | Mg | 10 |
| CDK2/CycA2 | MSA | Modified Histone H1 | 1000 | 27 | 25 | Mg | 5 |
| CDK2/CycE1 | MSA | Modified Histone H1 | 1000 | 130 | 150 | Mg | 5 |
| CDK3/CycE1 | MSA | Modified Histone H1 | 1000 | 1000 | 1000 | Mg | 5 |
| CDK4/CycD3 ^1)^ | MSA | DYRKtide-F | 1000 | 200 | 200 | Mg | 5 |
| CDK5/p25 | MSA | Modified Histone H1 | 1000 | 10 | 10 | Mg | 5 |
| CDK6/CycD3 ^1)^ | MSA | DYRKtide-F | 1000 | 330 | 300 | Mg | 5 |
| CDK7/CycH/MAT1 ^1)^ | MSA | CTD3 peptide | 1000 | 32 | 50 | Mg | 5 |
| CDK9/CycT1 ^1)^ | MSA | CDK9 substrate | 1000 | 9.4 | 10 | Mg | 5 |
| CGK2 ^3)^ | MSA | Kemptide | 1000 | 24 | 25 | Mg | 5 |
| CHK1 | MSA | CHKtide | 1000 | 50 | 50 | Mg | 5 |
| CHK2 | MSA | CHKtide | 1000 | 51 | 50 | Mg | 5 |
| CK1α ^1)^ | MSA | CKtide | 1000 | 4.1 | 5 | Mg | 5 |
| CK1γ1 | MSA | CKtide | 1000 | 6.3 | 5 | Mg | 5 |
| CK1γ2 | MSA | CKtide | 1000 | 10 | 10 | Mg | 5 |
| CK1γ3 | MSA | CKtide | 1000 | 3.2 | 5 | Mg | 5 |
| CK1δ | MSA | CKtide | 1000 | 7.7 | 10 | Mg | 5 |
| CK1ε ^1)^ | MSA | CKtide | 1000 | 16 | 25 | Mg | 5 |
| CK2α1/β | MSA | CK2tide | 1000 | 2.9 | 5 | Mg | 5 |
| CK2α2/β | MSA | CK2tide | 1000 | 2.1 | 5 | Mg | 5 |
| CLK1 | MSA | DYRKtide-F | 1000 | 11 | 10 | Mg | 5 |
| CLK2 | MSA | DYRKtide-F | 1000 | 140 | 150 | Mg | 5 |
| CLK3 | MSA | DYRKtide-F | 1000 | 75 | 75 | Mg | 5 |
| COT_Cascade | MSA | MAP2K1 | 1 | - | 1000 | Mg | 5 |
|  |  | Erk2 | 2.5 |  |  |  |  |
|  |  | Modified Erktide | 1000 |  |  |  |  |
| CRIK ^1)^ | MSA | Histone H3 peptide | 1000 | 7.8 | 10 | Mg | 5 |
| DAPK1 | MSA | DAPK1tide | 1000 | 1.1 | 1 | Mg | 5 |
| DCAMKL2 ^1)^ | MSA | GS peptide | 1000 | 120 | 150 | Mg | 5 |
| DLK_Cascade ^1)^ | MSA | MAP2K4 / MAP2K7 | 0.5 / 0.5 | - | 1000 | Mg | 5 |
|  |  | JNK2 | 50 |  |  |  |  |
|  |  | Modified Erktide | 1000 |  |  |  |  |
| DYRK1A | MSA | DYRKtide-F | 1000 | 16 | 25 | Mg | 5 |
| DYRK1B | MSA | DYRKtide-F | 1000 | 59 | 50 | Mg | 5 |
| DYRK2 | MSA | DYRKtide-F | 1000 | 7.7 | 10 | Mg | 5 |
| DYRK3 | MSA | DYRKtide-F | 1000 | 6.8 | 5 | Mg | 5 |
| EEF2K ^1)2)^ | MSA | EEF2Ktide | 1000 | 12 | 10 | Mg | 5 |
| Erk1 | MSA | Modified Erktide | 1000 | 34 | 50 | Mg | 5 |
| Erk2 | MSA | Modified Erktide | 1000 | 33 | 50 | Mg | 5 |
| Erk5 ^1)^ | MSA | EGFR-derived peptide | 1000 | 450 | 1000 | Mg | 5 |
| GSK3α | MSA | CREBtide-p | 1000 | 12 | 10 | Mg | 5 |
| GSK3β | MSA | CREBtide-p | 1000 | 9.1 | 10 | Mg | 5 |
| Haspin | MSA | Histone H3 peptide | 1000 | 140 | 150 | Mg | 5 |
| HGK | MSA | Moesin-derived peptide | 1000 | 9.4 | 10 | Mg | 5 |
| HIPK1 | MSA | DYRKtide-F | 1000 | 4.4 | 5 | Mg | 5 |
| HIPK2 | MSA | DYRKtide-F | 1000 | 5.9 | 5 | Mg | 5 |
| HIPK3 | MSA | DYRKtide-F | 1000 | 7.3 | 5 | Mg | 5 |
| HIPK4 | MSA | DYRKtide-F | 1000 | 7 | 5 | Mg | 5 |
| IKKα | IMAP | IκBα peptide | 100 | 41 | 40 | Mg | 10 |
| IKKβ | MSA | Modified IκBα-derived peptide | 1000 | 16 | 25 | Mg | 5 |
| IKKε ^1)^ | MSA | IκBα peptide | 1000 | 9.5 | 10 | Mg | 5 |
| IRAK1 | IMAP | SRPKtide | 100 | 27 | 25 | Mg | 2.5 |
| IRAK4 ^1)^ | MSA | IRAK1 peptide | 1000 | 917 | 1000 | Mg | 5 |
| JNK1 | MSA | Modified Erktide | 1000 | 29 | 100 | Mg | 5 |
| JNK2 | MSA | Modified Erktide | 1000 | 21 | 50 | Mg | 5 |
| JNK3 | MSA | Modified Erktide | 1000 | 6 | 25 | Mg | 5 |
| LATS2 ^1)^ | MSA | SGKtide | 1000 | 380 | 400 | Mg | 5 |
| LOK ^1)^ | MSA | Moesin-derived peptide | 1000 | 100 | 100 | Mg | 5 |
| MAP2K1_Cascade | MSA | Erk2 | 2.5 | - | 1000 | Mg | 5 |
|  |  | Modified Erktide | 1000 |  |  |  |  |
| MAP2K2_Cascade | MSA | Erk2 | 2.5 | - | 1000 | Mg | 5 |
|  |  | Modified Erktide | 1000 |  |  |  |  |
| MAP2K3_Cascade | MSA | p38α(9-352) | 10 | - | 1000 | Mg | 5 |
|  |  | Modified Erktide | 1000 |  |  |  |  |
| MAP2K4_Cascade ^1)^ | MSA | JNK2 | 50 | - | 1000 | Mg | 5 |
|  |  | Modified Erktide | 1000 |  |  |  |  |
| MAP2K5_Cascade ^1)^ | MSA | Erk5 | 50 | - | 1000 | Mg | 5 |
|  |  | EGFR-derived peptide | 1000 |  |  |  |  |
| MAP2K6_Cascade | MSA | p38α(9-352) | 10 | - | 1000 | Mg | 5 |
|  |  | Modified Erktide | 1000 |  |  |  |  |
| MAP2K7_Cascade ^1)^ | MSA | JNK2 | 50 | - | 1000 | Mg | 5 |
|  |  | Modified Erktide | 1000 |  |  |  |  |
| MAP3K1_Cascade | MSA | MAP2K1 | 1 | - | 1000 | Mg | 5 |
|  |  | Erk2 | 2.5 |  |  |  |  |
|  |  | Modified Erktide | 1000 |  |  |  |  |
| MAP3K2_Cascade ^1)^ | MSA | MAP2K4 / MAP2K7 | 0.5 / 0.5 | - | 1000 | Mg | 5 |
|  |  | JNK2 | 50 |  |  |  |  |
|  |  | Modified Erktide | 1000 |  |  |  |  |
| MAP3K3_Cascade | MSA | MAP2K6 | 1 | - | 1000 | Mg | 5 |
|  |  | p38α(9-352) | 10 |  |  |  |  |
|  |  | Modified Erktide | 1000 |  |  |  |  |
| MAP3K4_Cascade | MSA | MAP2K6 | 1 | - | 1000 | Mg | 5 |
|  |  | p38α(9-352) | 10 |  |  |  |  |
|  |  | Modified Erktide | 1000 |  |  |  |  |
| MAP3K5_Cascade | MSA | MAP2K6 | 1 | - | 1000 | Mg | 5 |
|  |  | p38α(9-352) | 10 |  |  |  |  |
|  |  | Modified Erktide | 1000 |  |  |  |  |
| MAP4K2 | MSA | S6k2 peptide | 1000 | 93 | 100 | Mg | 5 |
| MAPKAPK2 | MSA | GS peptide | 1000 | 3.6 | 5 | Mg | 5 |
| MAPKAPK3 | MSA | GS peptide | 1000 | 13 | 10 | Mg | 5 |
| MAPKAPK5 | MSA | GS peptide | 1000 | 12 | 10 | Mg | 5 |
| MARK1 | MSA | CHKtide | 1000 | 8 | 10 | Mg | 5 |
| MARK2 | MSA | CHKtide | 1000 | 8.8 | 10 | Mg | 5 |
| MARK3 | MSA | CHKtide | 1000 | 5 | 5 | Mg | 5 |
| MARK4 | MSA | CHKtide | 1000 | 12 | 10 | Mg | 5 |
| MELK ^1)^ | MSA | GS peptide | 1000 | 38 | 50 | Mg | 5 |
| MGC42105 | MSA | CHKtide | 1000 | 21 | 25 | Mg | 5 |
| MINK ^1)^ | MSA | Modified Erktide | 1000 | 36 | 50 | Mg | 5 |
| MLK1_Cascade | MSA | MAP2K1 | 1 | - | 1000 | Mg | 5 |
|  |  | Erk2 | 2.5 |  |  |  |  |
|  |  | Modified Erktide | 1000 |  |  |  |  |
| MLK2_Cascade | MSA | MAP2K1 | 1 | - | 1000 | Mg | 5 |
|  |  | Erk2 | 2.5 |  |  |  |  |
|  |  | Modified Erktide | 1000 |  |  |  |  |
| MLK3_Cascade | MSA | MAP2K1 | 1 | - | 1000 | Mg | 5 |
|  |  | Erk2 | 2.5 |  |  |  |  |
|  |  | Modified Erktide | 1000 |  |  |  |  |
| MNK1 | MSA | RS peptide | 1000 | 460 | 450 | Mg | 5 |
| MNK2 | MSA | RS peptide | 1000 | 110 | 100 | Mg | 5 |
| MOS_Cascade | MSA | MAP2K1 | 1 | - | 1000 | Mg | 5 |
|  |  | Erk2 | 2.5 |  |  |  |  |
|  |  | Modified Erktide | 1000 |  |  |  |  |
| MRCKα ^1)^ | MSA | DAPK1tide | 1000 | 0.45 | 1 | Mg | 5 |
| MRCKβ | MSA | DAPK1tide | 1000 | 0.67 | 1 | Mg | 5 |
| MSK1 | MSA | Crosstide | 1000 | 13 | 10 | Mg | 5 |
| MSK2 ^1)^ | MSA | Crosstide | 1000 | 40 | 50 | Mg | 5 |
| MSSK1 ^1)^ | MSA | DYRKtide-F | 1000 | 56 | 50 | Mg | 5 |
| MST1 ^1)10)^ | MSA | IRS1 | 1000 | 50 | 50 | Mg | 5 |
| MST2 ^1)6)^ | MSA | IRS1 | 1000 | 69 | 75 | Mg | 5 |
| MST3 ^1)^ | MSA | Moesin-derived peptide | 1000 | 66 | 75 | Mg | 5 |
| MST4 ^1)^ | MSA | Moesin-derived peptide | 1000 | 76 | 75 | Mg | 5 |
| NDR1 ^1)^ | MSA | SGKtide | 1000 | 12 | 10 | Mg | 5 |
| NDR2 ^1)^ | MSA | SGKtide | 1000 | 7.6 | 10 | Mg | 5 |
| NEK1 ^1)^ | MSA | CDK7 peptide | 1000 | 64 | 75 | Mg | 5 |
| NEK2 | MSA | CDK7 peptide | 1000 | 65 | 75 | Mg | 5 |
| NEK4 | MSA | GS peptide | 1000 | 51 | 50 | Mg | 5 |
| NEK6 ^1)^ | MSA | CDK7 peptide | 1000 | 69 | 75 | Mg | 5 |
| NEK7 ^1)^ | MSA | CDK7 peptide | 1000 | 40 | 50 | Mg | 5 |
| NEK9 ^1)^ | MSA | CDK7 peptide | 1000 | 190 | 200 | Mg | 5 |
| NuaK1 | MSA | CHKtide | 1000 | 59 | 50 | Mg | 5 |
| NuaK2 | MSA | CHKtide | 1000 | 26 | 25 | Mg | 5 |
| p38α | MSA | Modified Erktide | 1000 | 150 | 150 | Mg | 5 |
| p38β | MSA | Modified Erktide | 1000 | 63 | 75 | Mg | 5 |
| p38γ | MSA | Modified Erktide | 1000 | 13 | 10 | Mg | 5 |
| p38δ | MSA | Modified Erktide | 1000 | 5.8 | 5 | Mg | 5 |
| p70S6K | MSA | S6k2 peptide | 1000 | 14 | 10 | Mg | 5 |
| p70S6Kβ | MSA | S6k2 peptide | 1000 | 3.3 | 5 | Mg | 5 |
| PAK1 | MSA | LIMKtide | 1000 | 300 | 300 | Mg | 5 |
| PAK2 | MSA | DAPK1tide | 1000 | 81 | 100 | Mg | 5 |
| PAK4 ^1)^ | MSA | SGKtide | 1000 | 2.5 | 5 | Mg | 5 |
| PAK5 | MSA | DAPK1tide | 1000 | 1.9 | 1 | Mg | 5 |
| PAK6 ^1)^ | MSA | SGKtide | 1000 | 3.7 | 5 | Mg | 5 |
| PASK ^1)^ | MSA | GS peptide | 1000 | 9.7 | 10 | Mg | 5 |
| PBK ^1)^ | MSA | Histone H3 peptide | 1000 | 33 | 50 | Mg | 5 |
| PDHK2 ^1)^ | MSA | PDHKtide | 1000 | 28 | 25 | Mg+K | 5+3 |
| PDHK4 ^1)^ | MSA | PDHKtide | 1000 | 19 | 25 | Mg+K | 5+25 |
| PDK1 ^1)7)^ | MSA | T308tide | 1000 | 9.6 | 10 | Mg | 5 |
| PEK | IMAP | SRPKtide | 100 | 13 | 10 | Mg | 5 |
| PGK ^1)3)^ | MSA | Kemptide | 1000 | 8.2 | 10 | Mg | 5 |
| PHKG1 ^1)^ | MSA | GS peptide | 1000 | 71 | 75 | Mg | 5 |
| PHKG2 | MSA | GS peptide | 1000 | 8.1 | 10 | Mg | 5 |
| PIM1 | MSA | S6k2 peptide | 1000 | 640 | 500 | Mg | 5 |
| PIM2 ^1)^ | MSA | S6k2 peptide | 1000 | 4 | 5 | Mg | 5 |
| PIM3 | MSA | S6k2 peptide | 1000 | 130 | 150 | Mg | 5 |
| PKACα | MSA | Kemptide | 1000 | 2.6 | 5 | Mg | 5 |
| PKACβ | MSA | Kemptide | 1000 | 4.7 | 5 | Mg | 5 |
| PKACγ ^1)^ | MSA | Kemptide | 1000 | 4.5 | 5 | Mg | 5 |
| PKCα ^4)^ | MSA | PKC peptide | 1000 | 36 | 50 | Mg+Ca | 5+0.05 |
| PKCβ1 ^4)^ | MSA | PKC peptide | 1000 | 79 | 75 | Mg+Ca | 5+0.05 |
| PKCβ2 ^4)^ | MSA | PKC peptide | 1000 | 41 | 50 | Mg+Ca | 5+0.05 |
| PKCγ ^4)^ | MSA | PKC peptide | 1000 | 74 | 75 | Mg+Ca | 5+0.05 |
| PKCδ ^4)^ | MSA | PKC peptide | 1000 | 26 | 25 | Mg | 5 |
| PKCε ^4)^ | MSA | PKC peptide | 1000 | 16 | 25 | Mg | 5 |
| PKCζ | MSA | PKC peptide | 1000 | 11 | 10 | Mg | 5 |
| PKCη ^4)^ | MSA | PKC peptide | 1000 | 36 | 50 | Mg | 5 |
| PKCθ ^4)^ | MSA | PKC peptide | 1000 | 25 | 25 | Mg | 5 |
| PKCι | MSA | PKC peptide | 1000 | 24 | 25 | Mg | 5 |
| PKD1 | MSA | GS peptide | 1000 | 25 | 25 | Mg | 5 |
| PKD2 | MSA | GS peptide | 1000 | 26 | 25 | Mg | 5 |
| PKD3 | MSA | GS peptide | 1000 | 34 | 50 | Mg | 5 |
| PKN1 | IMAP | S6K peptide | 100 | 19 | 25 | Mg | 1 |
| PKR | IMAP | SRPKtide | 100 | 13 | 10 | Mg | 5 |
| PLK1 ^1)^ | MSA | CDC25ctide | 1000 | 5.6 | 5 | Mg | 5 |
| PLK2 | IMAP | CHK2 peptide | 50 | 30 | 30 | Mg | 10 |
| PLK3 | MSA | CDC25ctide | 1000 | 6.8 | 5 | Mg | 5 |
| PRKX ^1)^ | MSA | Kemptide | 1000 | 20 | 25 | Mg | 5 |
| QIK | MSA | AMARA peptide | 1000 | 42 | 50 | Mg | 5 |
| RAF1_Cascade | MSA | MAP2K1 | 1 | - | 1000 | Mg | 5 |
|  |  | Erk2 | 2.5 |  |  |  |  |
|  |  | Modified Erktide | 1000 |  |  |  |  |
| ROCK1 | MSA | LIMKtide | 1000 | 3.1 | 5 | Mg | 5 |
| ROCK2 | MSA | LIMKtide | 1000 | 7.4 | 5 | Mg | 5 |
| RSK1 | MSA | S6K peptide (N-FL) | 1000 | 21 | 25 | Mg | 5 |
| RSK2 | MSA | S6K peptide (N-FL) | 1000 | 14 | 10 | Mg | 5 |
| RSK3 | MSA | S6K peptide (N-FL) | 1000 | 9.9 | 10 | Mg | 5 |
| RSK4 | MSA | S6K peptide (N-FL) | 1000 | 20 | 25 | Mg | 5 |
| SGK | MSA | SGKtide | 1000 | 52 | 50 | Mg | 5 |
| SGK2 | MSA | SGKtide | 1000 | 58 | 50 | Mg | 5 |
| SGK3 | MSA | SGKtide | 1000 | 17 | 25 | Mg | 5 |
| SIK ^1)^ | MSA | AMARA peptide | 1000 | 47 | 50 | Mg | 5 |
| skMLCK ^2)^ | MSA | MLCtide | 1000 | 820 | 1000 | Mg | 5 |
| SLK ^1)^ | MSA | Moesin-derived peptide | 1000 | 36 | 50 | Mg | 5 |
| SRPK1 | IMAP | SRPKtide | 100 | 200 | 100 | Mg | 10 |
| SRPK2 ^1)^ | MSA | DYRKtide-F | 1000 | 14 | 10 | Mg | 5 |
| TAK1-TAB1_Cascade ^1)^ | MSA | MAP2K4 / MAP2K7 | 0.5 / 0.5 | - | 1000 | Mg | 5 |
|  |  | JNK2 | 50 |  |  |  |  |
|  |  | Modified Erktide | 1000 |  |  |  |  |
| TAOK2 ^1)6)^ | MSA | TAOKtide | 1000 | 39 | 50 | Mg | 5 |
| TBK1 | MSA | CKtide | 1000 | 21 | 25 | Mg | 5 |
| TNIK | MSA | Moesin-derived peptide | 1000 | 16 | 25 | Mg | 5 |
| TSSK1 | MSA | GS peptide | 1000 | 11 | 10 | Mg | 5 |
| TSSK2 ^1)^ | MSA | GS peptide | 1000 | 8.8 | 10 | Mg | 5 |
| TSSK3 ^1)^ | MSA | GS peptide | 1000 | 45 | 50 | Mg | 5 |
| WNK1 ^1)^ | MSA | SPAKtide | 1000 | 140 | 150 | Mg+Mn | 5+3 |
| WNK2 ^1)^ | MSA | SPAKtide | 1000 | 48 | 50 | Mg+Mn | 5+3 |
| WNK3 ^1)^ | MSA | SPAKtide | 1000 | 48 | 50 | Mg+Mn | 5+3 |
| PIK3CA/PIK3R1 ^1)8)^ | MSA | Phosphatidyl- inositol | 1000 | 58 | 50 | Mg | 5 |
| SPHK1 | MSA | Sphingosine | 1000 | 20 | 25 | Mg | 5 |
| SPHK2 | MSA | Sphingosine | 1000 | 620 | 600 | Mg | 5 |

^1)^ Reaction time is 5 hours.

^2)^ CaCl_2_, Calmodulin are added at the final concentration of 1 mM and 10 μg/ml, respectively.

^3)^ cGMP is added at the final concentration of 5 μM.

^4)^ Phosphatidylserine and Diacyl Glycerol are added at the final concentration of 50 μg/mL and 5 μg/mL, respectively.

^5)^ Sodium orthovanadate is added at the final concentration of 25 μM.

^6)^ Cantharidin is added at the final concentration of 10 μM.

^7)^ PIFtide and Cantharidin are added at the final concentration of 2 μM and 20 μM, respectively.

^8)^ Assay buffer is 20 mM HEPES(pH 7.5), 2mM DTT. Sodium cholate, NaCl and cantharidine are added at the final concentration of 25 μM, 75 mM and 20 μM, respectively.

^9)^ TPX2 peptide is added at the final concentration of 200 nM.

^10)^ Cantharidin is added at the final concentration of 20 μM.

**Supplementary Methods**

**Chemical Synthesis**

**General notes:** Analytical thin-layer chromatography (TLC) was performed on precoated (0.25 mm) silica-gel plates (Merck Chemicals, Silica Gel 60 F_254_, Cat. No. 1.05715). Preparative thin-layer chromatography (PTLC) was performed on silica-gel (Wako Pure Chemical Industries Ltd., Wakogel B5-F, Cat. No. 230-0043). Melting point (Mp) was measured on an OptiMelt MPA100 automated melting point apparatus (Stanford Research Systems), and is uncorrected. IR spectrum was measured by diffuse reflectance method on a Shimadzu IRPrestige-21 spectrometer attached with DRS-8000A with the absorption band given in cm^–1^. ^1^H and ^13^C NMR spectra were obtained with a Bruker AVANCE 500 spectrometer at 500 and 126 MHz, respectively. CDCl_3_ containing 0.03% tetramethylsilane (99.8%D, Kanto Chemical Co. Inc., Cat. No. 07663-23) was used as a solvent for obtaining NMR spectra. Chemical shifts (δ) are given in parts per million (ppm) downfield from (CH_3_)_4_Si (δ 0.00) as an internal reference with coupling constants (*J*) in hertz (Hz). The abbreviations s and d signify singlet and doublet, respectively. High-resolution mass spectrum (HRMS) was measured on a Bruker micrOTOF mass spectrometer under positive electrospray ionization (ESI^+^) conditions.

5-(4-Pyridinyl)-1*H*-indazole (**TG693**)

Under argon atmosphere, a suspension of 5-bromoindazole (118 mg, 0.601 mmol), 4-pyridylboronic acid (110 mg, 0.898 mmol), Pd_2_(dba)_3_ (27.5 mg, 30.0 μmol), XPhos (57.6 mg, 0.118 mmol), and K_3_PO_4_·*n*H_2_O (255 mg, <1.20 mmol) in *n*-BuOH (2.4 mL) was stirred for 24 h at 100 °C (oil bath temperature). After cooling to room temperature, the mixture was passed through a short pad of Celite and concentrated under reduced pressure. The obtained orange oil was purified by preparative TLC (CHCl_3_/MeOH = 10/1) to give **TG693** (24.4 mg, 0.125 mmol, 20.8%) as a yellow solid.

TLC *R*_f_ = 0.26 (CH_2_Cl_2_/MeOH = 20/1); Mp 190 ºC (decomp.); IR (cm^–1^) 792, 798, 802, 1030, 1349, 1420, 1490, 1603, 3361; ^1^H NMR (CDCl_3_, 500 MHz) δ 7.57 (AA**´**BB**´**, 2H), 7.62 (d, *J* = 8.5 Hz, 1H), 7.70 (dd, *J* = 1.3, 8.5 Hz, 1H), 8.06 (d, *J* = 1.3 Hz, 1H), 8.18 (s, 1H), 8.68 (AA**´**BB**´**, 2H) (The signal for N*H* of indazole was not observed); ^13^C NMR (CDCl_3_, 126 MHz) δ 110.5, 119.7, 121.4, 121.8, 123.9, 126.3, 131.5, 135.6, 140.2, 150.1; HRMS (ESI^+^) *m*/*z* 196.0867 ([M+H]^+^, C_12_H_10_N_3_ requires 196.0869).

**Single dose oral toxicity studies in rats**

Male and female 4-week-old Crl: CD (SD) rats were purchased from Charles River Laboratories Inc. After starvation for 6 h, animals were orally administrated with TG693 (100 mg kg^-1^) or the vechicle (0.5% methylcellulose) alone. Gross appearances of animals were observed immediately and after 6 h of the administration and thereafter once a day for 7 days. Body weights were measured at 0, 1, 3, 7 days after oral administration.
